# SMARCB1-driven EGFR-GLI1 epigenetic alterations in lung cancer progression and therapy are differentially modulated by MEOX2 and GLI-1

**DOI:** 10.1038/s41417-025-00873-0

**Published:** 2025-02-19

**Authors:** Octavio A. Trejo-Villegas, Priscila Pineda-Villegas, Leonel Armas-López, Criselda Mendoza-Milla, Irlanda Peralta-Arrieta, Oscar Arrieta, Irene H. Heijink, Joaquín Zúñiga, Federico Ávila-Moreno

**Affiliations:** 1 Lung Diseases and Functional Epigenomics Laboratory (LUDIFE), Biomedicine Research Unit (UBIMED), Facultad de Estudios Superiores-Iztacala (FES-Iztacala), Universidad Nacional Autónoma de México, (UNAM), Avenida de los Barrios #1, Colonia Los Reyes Iztacala, Tlalnepantla de Baz, México; 2 Research Unit, Instituto Nacional de Enfermedades Respiratorias (INER), Ismael Cosío Villegas, Ciudad de México, México; 3 Thoracic Oncology Unit, Instituto Nacional de Cancerología (INCan), Ciudad de México, México; 4 University of Groningen, Departments of Pathology & Medical Biology and Pulmonology, GRIAC Research Institute, University Medical Center Groningen, Groningen, Netherlands; 5 Tecnologico de Monterrey, Escuela de Medicina y Ciencias de la Salud, Ciudad de México, México; 6 Research Tower, Subdirección de Investigación Básica, Instituto Nacional de Cancerología (INCan), Ciudad de México, México

**Keywords:** Non-small-cell lung cancer, Cancer therapeutic resistance, Cancer genetics, Oncogenes

## Abstract

Lung cancer remains the leading cause of cancer-related mortality globally, with genes such as SMARCB1, MEOX2, and GLI-1 playing significant roles in its malignancy. Despite their known involvement, the specific molecular contributions of these genes to lung cancer progression, particularly their effects on epigenetic modifications on oncogenes sequences as EGFR and GLI-1, and their influence in the response to EGFR-TKI-based therapies, have not been fully explored. Our study reveals how MEOX2 and GLI-1 are key molecular modulators of the GLI-1 and EGFR-epigenetic patterns, which in turn transcriptionally and epigenetically affect EGFR gene expression in lung cancer. Additionally, MEOX2 was found to significantly promote in vivo lung tumor progression and diminish the effectiveness of EGFR-TKI therapies. Conversely, mSWI/SNF derived subunit SMARCB1 was detected to suppress tumor growth and enhance the oncological therapeutic response in in vivo studies by inducing epigenetic modifications in the GLI-1 and EGFR genetic sequences. Furthermore, our results suggest that BRD9 may contribute to the activation of both lung cancer oncogenes GLI-1 and EGFR. Such findings suggest that SMARCB1 and MEOX2 could serve as important prognosis biomarkers and target genes in human lung cancer therapy, offering new opportunities for the development of more effective and selective treatment strategies in the field of lung malignant diseases.

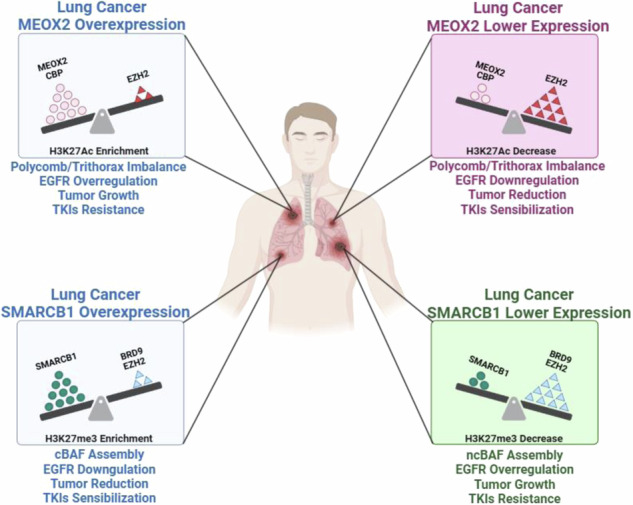

## Introduction

Lung cancer is the leading cause of cancer-related deaths worldwide, with a five-year survival rate of less than 20% after diagnosis [1]. Within the molecular complex landscape in human lung malignant diseases, recently a significant gene of interest is MEOX2, this gene is responsible for encoding a transcription factor linked to homeobox activity, playing a crucial role in embryonic development, tissue repairing, and regeneration processes [2, 3]. Oncology research indicates how MEOX2 influences lung cancer pathogenesis, demonstrating its influence on key cancer hallmarks, including cellular proliferation, invasion, metastasis, angiogenesis, and drug resistance. Furthermore, MEOX2 has recently been proposed as a molecular regulator of GLI-1 oncogene expression, which is the final effector of the Sonic Hedgehog cell signaling pathway. Reports have associated GLI-1 with enhanced proliferation, inhibition of apoptosis, metastasis, poor survival outcomes, and chemoresistance in lung cancer [4–8]. Moreover, experimental studies indicate that the MEOX2 and GLI-1 transcription factors can influence the genetic expression of EGFR, a key driver gene of non-small cell lung carcinomas (NSCLC), by modulating specific histone marks on its enhancer and promoter sequences. MEOX2 and GLI-1 affect the molecular balance between repressive H3K27me3 and activating H3K27Ac histone marks in NSCLC cells. Additionally, this regulation involves chromatin remodeling proteins, such as SMARCB1, which binds to EGFR gene regulatory sequences, further shaping its expression and contributing to lung cancer progression [8]. Given the critical role of EGFR in NSCLC, EGFR-targeted tyrosine kinase inhibitors (TKIs) have become essential therapies. These agents, including erlotinib, afatinib and others, have gained FDA approval for treating NSCLC with specific EGFR mutations, offering targeted treatment options that improve patient outcomes. However, EGFR-TKI efficacy in wild-type EGFR (EGFR-WT) NSCLC remains limited, partly because the mechanisms behind resistance in these lung malignant cases are not fully understood [9–11]. Intriguingly, studies suggest that SMARCB1, a recognized tumor suppressor gene in various cancers, may play a significant role influencing sensitivity to EGFR-TKI-based therapy in malignant rhabdoid tumors (MRT). Nonetheless, despite its significant involvement in different malignancies, the specific function of SMARCB1 in lung cancer remains underexplored [12–17]. On that note, SMARCB1 as a subunit of the switch/sucrose non-fermentable (SWI/SNF) protein complex, acts as a part of the Trithorax protein group. Such complex is known for its chromatin remodeling activity, which has been implicated in numerous human malignancies. Mammalian SWI/SNF (mSWI/SNF) complexes, comprised of 29 codifying genes, categorized into three subfamilies known as canonical BAF (cBAF), Polybromo-associated BAF (PBAF), and non-canonical BAF (ncBAF), each of these three complexes contains unique subunits contributing to their specific functional characteristics and molecular specificity [18, 19]. Remarkably, genes that encode subunits of the mSWI/SNF complexes have been detected to display notably high mutation rates in diverse human malignancies [20], including human lung malignancies [21, 22], and several lung non-malignant diseases [23]. Research findings have suggested that ncBAF assembly, characterized by the loss of SMARCB1 and the incorporation of BRD9, may exhibit oncogenic properties by activating oncogenes and enhancing cell proliferation [24–27]. Notably, BRD9 has been reported to be upregulated in solid NSCLC and NSCLC cell lines. This upregulation has been associated with enhanced cellular proliferation, migration, and invasion capabilities in NSCLC [28, 29]. Moreover, in squamous cell lung carcinomas, BRD9 enhances cellular proliferation by positively regulating the expression of the MYC oncogene [30]. Nonetheless, the impact of SMARCB1 loss on the activation of oncogenes in human lung cancer, particularly through the assembly of the ncBAF complex via BRD9 and other chromatin remodelers, as well as the potential roles of MEOX2, GLI-1, and SMARCB1 in response to EGFR-TKI therapies and clinical outcomes in lung cancer patients, has not compressively been understood.

## Methods

### Human lung cancer cell lines and cellular cultures

The A549 and NCI-H1975 human NSCLC cell lines were purchased from ATCC (University Boulevard, USA). The cells were grown in RPMI-1640 medium with 10% fetal bovine serum and 1% ampicillin/streptomycin (Biowest, USA) at 37 °C in a humidified atmosphere with 5% CO_2_.

### Short hairpin RNAs (shRNAs) silencing assays

A549 and NCI-H1975 cells were seeded in six-well plates until 80% confluence in antibiotic-free RPMI-1640 (Biowest, USA) for 16 h. Transfection solutions were prepared following the instructions provided by the supplier with plasmids for MEOX2, GLI-1 and SMARCB1 and a scrambled (SCR) sequence (Santa Cruz Biotechnology, USA) as a negative control. A 200 µL aliquot of the prepared transfection solution was added to 800 µL of transfection medium, resulting in a final volume of 1 mL per well. Cells were incubated with the transfection medium under standard growth conditions for 6 h, after which 1 mL of RPMI-1640 medium, supplemented with twice the standard concentrations of serum and antibiotics, was added to each well. Cells were then maintained for an additional 24 h under normal growth conditions. Following this incubation, transfected cells were subjected to selection with puromycin (Sigma Aldrich, St. Louis, MO), beginning at an initial concentration of 0.5 µg/mL in standard growth medium. Selection medium was refreshed every 48 h, with the puromycin concentration gradually increased to 5 µg/mL to establish a stable transfection model. Cells were then maintained in medium containing 5 µg/mL puromycin for subsequent assays. The transfected cells were named shSCR, shMEOX2, shGLI-1 and shSMARCB1.

### RNA isolation and RT-qPCR assays

Total RNA was isolated from Cell cultures (*n* = 3) and tumors (*n* = 2) under different conditions using TRIzol (Invitrogen, USA) as per the manufacturer’s instructions. RNA concentration was evaluated using a UV/VIS spectrophotometer (Fisher Scientific, USA). One microgram of total RNA was used for reverse transcription with the RevertAid H Minus First Strand cDNA Synthesis Kit (Fisher Scientific, USA). mRNA expression profiles were determined by qPCR using 20 ng of cDNA and SYBR Green (AMPLIQON, Denmark). The qPCR conditions were: initial denaturation at 95 °C for 15 min, 40 cycles at 95 °C for 30 s and 60 °C for 1 min. Melt curve: 95 °C for 5 min, 65 °C for 1 min, and 97 °C continuous. qPCR was performed on the LightCycler 480 System (Roche, USA). Relative quantification was calculated using the 2^−^^ΔΔCt^ method with GAPDH as the control. All reactions were in triplicates. qPCR primers are listed in Table S1.

### Western blot

Cell cultures (*n* = 3) and tumors (*n* = 2) under different conditions were washed in PBS and lysed in RIPA buffer (HEPES-KOH 1 M pH 7.5, NaCl 5 M, EDTA 0.5 M pH 8, Triton X-100 20%, Sodium Deoxycholate 10%, SDS 20%) with protease and phosphatase inhibitors (Roche, USA). Proteins were quantified using Bio-Rad DC™ Protein Assay kit (Bio-Rad, USA), and absorbance was determined at 750 nm (BioTek Instruments, USA). For western blot analysis, 30 μg protein were separated on 10% SDS/PAGE, transferred to PVDF membrane, blocked in TBS-T with 5% milk, incubated with primary antibodies overnight at 4 °C, washed, and incubated with secondary antibodies. Antibody details are in Table S2. Proteins were visualized using chemiluminescence detection reagent (Clarity™ Western ECL Substrate; Bio-Rad, USA). GAPDH was used as a loading control. Protein levels were analyzed using IMAGEJ software.

### Cellular viability (MTT assays)

TKI-afatinib (Cayman Chemical, USA) was solubilized in DMSO as a 10 mM stock solution. shA549 cells (shSCR, shSMARCB1) were seeded in 96-well plates with RPMI-1640 containing 10% FBS for overnight attachment. Cells were then treated with TKI-afatinib at serial dilutions (0.001–1000 μM). Control cells were cultured in complete medium with DMSO, maintaining a final DMSO concentration of 0.1–2.5%. Cell viability was assessed through MTT assays by adding 5 mg/mL MTT solution (Sigma Aldrich, USA) to each well after 72 h of incubation at 37 °C. After a 4-h incubation, the medium was removed, and crystals were dissolved in DMSO. Absorbance was measured at 570 nm. Inhibitory concentrations (IC50) were calculated using GraphPad Prism software 8.0 (GraphPad Software, USA). Biological replicates, *n* = 3.

### In vivo lung tumor progression

shA549 cells (shSCR, shMEOX2, shGLI-1, and shSMARCB1 groups) were cultured under standard conditions as previously described. Six-week-old male nu-/nu- mice, bred under pathogen-free conditions, were obtained from INCMNSZ. The mice were housed in groups of 4–5 per cage with unrestricted access to food and water and were allowed to acclimate to the facility for two weeks. At eight weeks of age, mice were randomly assigned to experimental groups (*n* = 4) to ensure even distribution of baseline characteristics, including body weight and overall health. The sample size for the mouse experiments was determined in accordance with established guidelines [31, 32]. Each mouse received a subcutaneous injection of 5 × 10^6^ shA549 cells (0.1 mL per injection). Following injection, tumor growth was carefully monitored, once tumors became palpable, tumor progression was assessed with a focus on the impact of our genes of interest on tumor development dynamics. Mice were then reassigned into either vehicle or treatment groups through a secondary randomization process (Days 30–33). Although groups were not specifically stratified by tumor size before initiating TKI treatment, this approach allowed us to capture a more representative range of tumor growth rates. This choice preserves the natural variability in tumor development, avoiding potential biases that could arise from stratifying by tumor size and enhancing the generalizability of the treatment effects observed across heterogeneous tumor presentations. For treatment, mice received either TKI-erlotinib (Sigma Aldrich, USA) or TKI-afatinib (Cayman Chemical, USA), dissolved in a solution of 10% DMSO, 10% Tween 80, and 0.5% hydroxypropyl-methylcellulose (Sigma Aldrich, USA) to facilitate intraperitoneal administration. Treatments were administered daily at a dose of 10 mg/kg over seven days. Tumor volumes were measured using calipers and calculated with the formula V = (3.1416/6)*(L*W*H) where L, W, and H represent tumor length, width, and height, respectively. Mice were monitored daily for health and signs of distress. Humane endpoints were predefined to include tumor size exceeding 1500 mm³, body weight loss of more than 15%, and clinical signs of distress, including vomiting, skin ulceration, or impaired mobility. Mice reaching these endpoints were euthanized to prevent unnecessary suffering. All mice were euthanized with pentobarbital sodium at the end of the experiment, and tumors were stored at −80 °C.

### ChIP-qPCR assays

The solid in vivo lung tumors from the shA549 groups were pulverized using a BioPulverizer (BioSpec, USA) in liquid nitrogen. The resulting material was resuspended in cold 1x PBS, crosslinked with 1% formaldehyde, and neutralized with glycine. The pulverized tumor was centrifuged, washed with 1x PBS, and treated with cell lysis buffer containing protease inhibitors (Roche, USA). The chromatin was sonicated for 10 pulses of 20 s at 60 watts. Then, 20 μg of fragmented chromatin was immunoprecipitated using a commercial EZ-Magna ChIP^TM^ G kit (Millipore, USA) with one μg of the antibodies of interest. One microgram of IgG was used as a negative control (Millipore, USA). The IP-DNAs (*n* = 2 for each antibody in each condition) were analyzed by absolute quantification via qPCR. Twenty nanograms of IP-DNA were used per reaction, with amplification of standard curves through serial dilutions (100–0.01 ng) of diploid control genomic DNA from healthy donor peripheral blood mononuclear cells. Amplification conditions were: initial denaturation at 95 °C for 10 min, 60 cycles of 95 °C for 15 s, annealing at 55 °C for 30 s, and extension at 72 °C for 30 s. Oligonucleotides for the EGFR, GLI-1, SMARCB1, and EZH2 genetic enhancer and promoter sequences were designed with Primer3Plus and synthesized by T4OLIGO Company (Mexico).

### Progression-free disease interval analysis

We accessed The Cancer Genome Atlas (TCGA) Lung Cancer dataset for patients with lung cancer treated with TKIs and gene expression data. We constructed progression-free interval (PFI) curves using Xenabrowser (University of California, Santa Cruz; https://xenabrowser.net/) and compared them using the Log-Rank method.

### Co-immunoprecipitation (Co-IP) assays

The A549 parental cells were crosslinked with 1% formaldehyde, neutralized with glycine, rinsed with 1x PBS, detached by scraping, centrifuged, and treated with Co-IP buffer (SDS 1%, 10 mM EDTA, 50 mM Tris-HCl). Chromatin was sonicated, immunoprecipitated, and analyzed by western blot according to standard protocols. Loading of 10% of Input was performed as a positive control and the immunoprecipitation of the IgG antibody was used as a negative control.

### STRING analysis

The protein-protein interaction network for MEOX2, SMARCB1, GLI-1, EZH2, CBP, and BRD9 was analyzed using the STRING (Search Tool for the Retrieval of Interacting Genes/Proteins) database (version 12.0) with an interaction confidence threshold set to 0.400 (http://www.string-db.org/).

### Statistical analysis

Data are presented as mean ± S.D. Differences between the two groups were assessed using unpaired *t-*test (two-tailed). Statistical analysis was carried out using GraphPad Prism 8.0. Significance was defined as *P*-values: *P < 0.05, **P < 0.01; ***P < 0.001, and ****P < 0.0001, to enhance the clarity of the graphs, only significant *P*-values are displayed. The absence of an asterisk indicates a *P*-value greater than 0.05. The variance within each group was assessed by calculating the standard deviation, and comparable variances between groups were verified prior to performing statistical analysis.

## Results

### MEOX2 promotes greater lung tumor progression in vivo and resistance to EGFR-TKI therapy compared to GLI-1

**Fig. 1:**
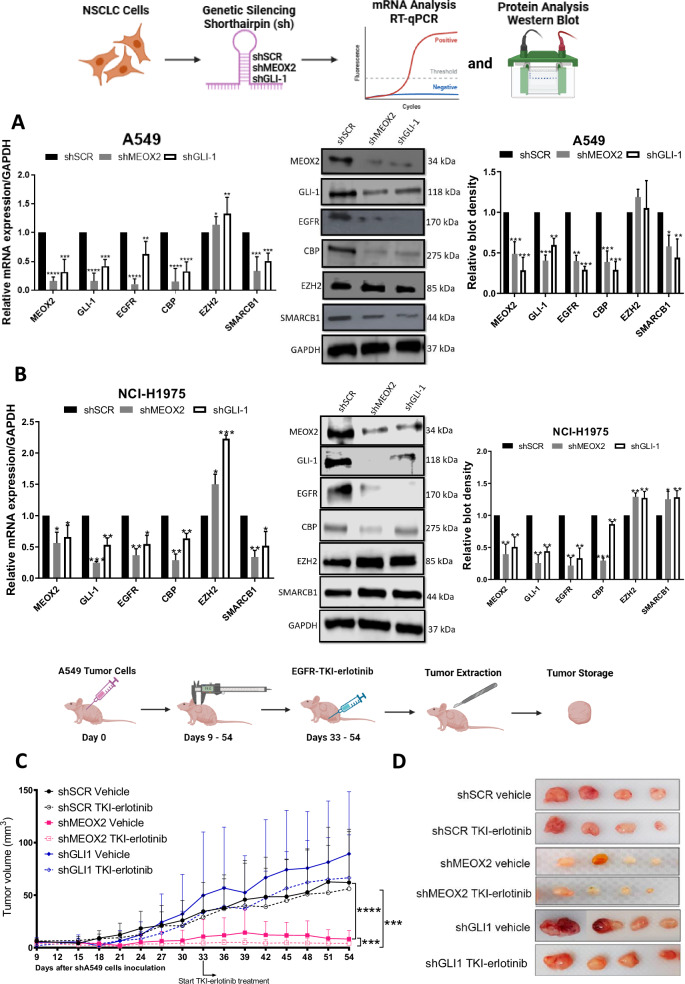

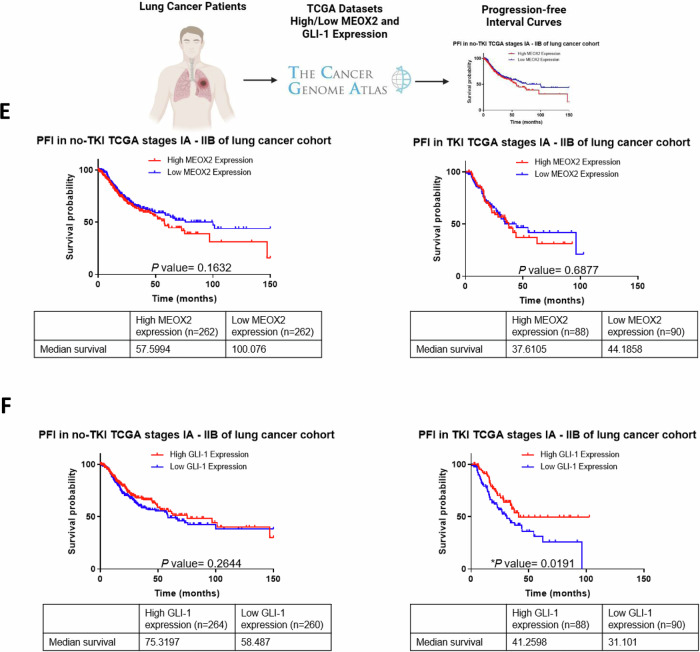
Reduced expression of MEOX2/GLI-1 in human lung cancer cells reduces in vivo xenotransplanted tumoral progression and is associated with an increased PFI in both non-treated and EGFR-TKI-treated lung cancer patients. **A** Cellular cultures of monolayer A549 lung cancer cells show that MEOX2 and GLI-1 were effectively silenced by shRNAs, triggering a decrease in CBP, EGFR and SMARCB1 and an increase in EZH2 mRNA expression and protein abundance. Data represent the mean ± S.D. of three independent biological replicates. **B** H1975 cells show that MEOX2 and GLI-1 were silenced by shRNAs, triggering changes in EGFR, CBP, EZH2 and SMARCB1 mRNA expression and protein levels. Data represents the mean ± S.D. of three independent biological replicates. **C** Analysis of tumor volume in immuno-deficient nu-/nu- mice was conducted in shSCR, shMEOX2, and shGLI-1 groups under vehicle (10% DMSO, 10% Tween 80, 0.5% hydroxypropyl-methylcellulose) and TKI-erlotinib (10 mg/kg) treatments. Tumor volume was measured twice weekly, represented as mean ± S.D. for 4 mice per group. Graph shows one experiment of two replicates. **D** Visual representation of tumor growth at the end of in vivo progression, showing one experiment (*n* = 4 tumors) of two replicates. **E** Analysis of the clinical progression-free disease interval (PFI) for MEOX2-associated expression in earlier clinical stages (IA-IIB) of non-TKI-treated lung cancer patients, and PFI for MEOX2-associated expression in earlier clinical stages (IA-IIB) of TKI-treated lung cancer patients. **F** PFI analysis for GLI-1-associated expression in earlier clinical stages (IA-IIB) of non-TKI-treated lung cancer patients, and PFI for GLI-1-associated expression in earlier clinical stages (IA-IIB) of TKI-treated lung cancer patients.

To investigate MEOX2 and GLI-1 roles in lung tumor progression, we conducted a genetic silencing experiment (shRNAs) targeting these genes using A549 and NCI-H1975 NSCLC cells and analyzed mRNA expression and protein levels of the EGFR oncogene, as well as key epigenetic markers from the Trithorax group, including CBP and SMARCB1, and EZH2, a member of the Polycomb repressive complex. Results showed a significant decrease in EGFR, CBP, and SMARCB1 at mRNA and protein levels, and a significant increase in mRNA of EZH2, but no significant change in protein levels for EZH2, suggesting that both MEOX2/GLI-1 modulate these tumor and epigenetic markers in lung cancer A549 cells (Fig. 1A). In addition, similar results were obtained on the NCI-H1975 NSCLC cells, highlighting the significant decrease in EGFR, CBP, and SMARCB1 mRNA levels, but a significant increase in EZH2 at mRNA and protein levels, as well as SMARCB1 protein levels. Notably, the increase of SMARCB1 protein levels contrasts with the findings observed in the lung cancer A549 cells (Fig. 1B). The finding that MEOX2 and GLI-1 modulate the protein levels of the EGFR oncogene, combined with previous reports linking MEOX2 and GLI-1 to proliferation control in lung cancer prompted us to investigate in vivo lung cancer progression (nu-/nu- mice), and response to EGFR-TKIs, specifically focusing on the effects of the TKI-erlotinib on the erlotinib-resistant lung cancer A549 cell line. This choice was driven by the current lack of established methods for sensitizing an EGFR-WT cell line to erlotinib. This analysis demonstrated a significant reduction in tumor size in the shMEOX2 group, while, unexpectedly, a non-significant increase in tumor size was observed in the shGLI-1 group compared to the shSCR control group. TKI-erlotinib treatment significantly reduced tumor size in the shMEOX2 group, while a non-significant decrease was observed in the shGLI-1 experimental group. These results confirm that both MEOX2/GLI-1 play a pivotal role in regulating EGFR and suggest that MEOX2 is mediating the response to EGFR-TKIs-based therapy (Fig. 1C, D). To establish a connection between these findings and lung cancer patients, we examined a patient cohort from the TCGA Lung Cancer dataset. Analysis of clinical outcomes revealed a non-significant trend indicating that higher MEOX2 expression may be associated with shorter PFI in early clinical stages. This trend was observed in both non-TKI-treated patients (57.59 vs. 100.07 months) and TKI-treated patients (37.61 vs. 44.18 months). However, higher GLI-1 expression was associated with a non-significant trend toward extended PFI in non-TKI-treated patients (75.31 vs. 58.48 months), and also a significant extended PFI in TKI-treated lung cancer patients (41.25 vs. 31.10 months), showing that our in vivo model demonstrated a trend that diverges from the tendency observed in lung cancer patients with low MEOX2 versus low GLI-1 expression (Fig. 1E, F). Additionally, no significant associations were observed between MEOX2 or GLI-1 expression and PFI in patients with advanced-stage lung cancer (Fig. S1). Overall, these results suggest that MEOX2 and GLI-1 play divergent roles in lung cancer progression, and response to EGFR-TKIs-based therapy, with both MEOX2/GLI-1 potentially influencing EGFR gene expression.

### MEOX2 and GLI-1 drive lung tumor progression with EZH2/H3K27me3, CBP/H3K27Ac and SMARCB1 epigenetic imbalance

**Fig. 2:**
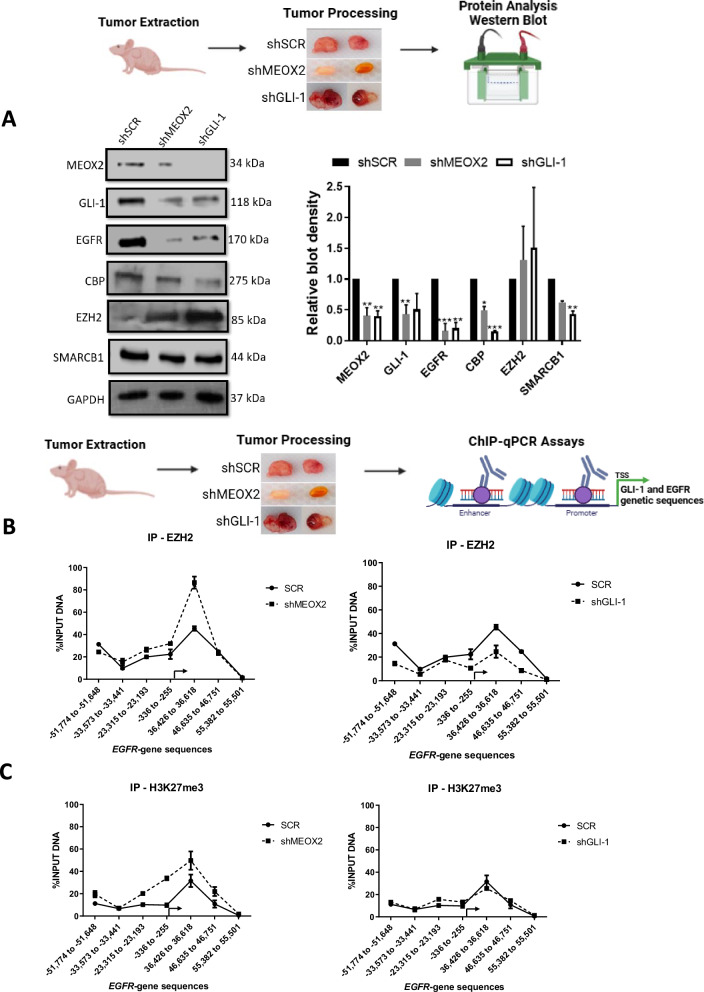

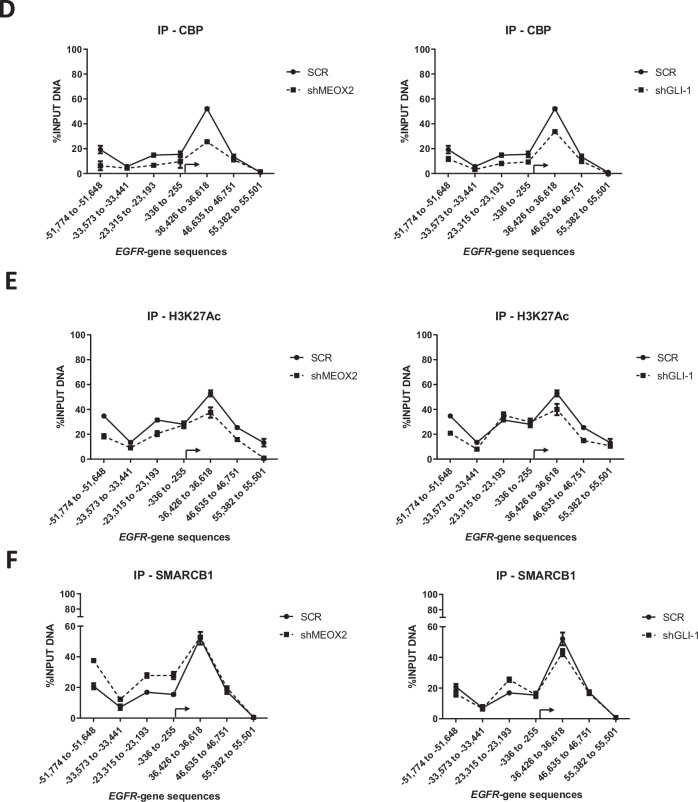
Genetic silencing assays of the MEOX2/GLI-1 reduce EGFR-gene expression, altering epigenetic marks EZH2/H3K27me3, and reducing CBP/H3K27Ac on EGFR-gene sequences*.* **A** In vivo lung tumors show that genetic silencing of MEOX2/GLI-1 reduces EGFR, CBP and SMARCB1 but increases EZH2 protein abundance. GAPDH was used as loading control. Data represents the mean ± S.D. of two independent biological replicates. **B** The occupation of the histone methyltransferase, EZH2, **C** histone H3K27me3, **D** histone acetyltransferase, CBP **E** histone H3K27Ac and **F** SWI/SNF member-SMARCB1 were quantified by the effect of genetic silencing of MEOX2/GLI-1 at super-enhancer and promoter sequences of the *EGFR*-gene, in lung tumors. Graph shows a single representative experiment of two biological replicates.

Given that the low expression of MEOX2 and GLI-1 was associated with improved responses to EGFR-TKIs using xenograft in vivo models, we further investigated whether activated or repressive epigenetic marks might regulate EGFR expression through an in vivo study. As a result, we assessed the protein levels from solid in vivo lung tumors derived from shSCR, shMEOX2, and shGLI-1 experimental groups under the experimental Vehicle condition (Fig. 1D). Our analysis revealed a significant decrease in the protein levels of EGFR, CBP, and SMARCB1 under shMEOX2 and shGLI-1 conditions, while a non-significant increase is observed in EZH2 protein levels. Indicating how MEOX2 and GLI-1 regulate the abundance of oncogenic and epigenetic markers through lung cancer in vivo progression, suggesting their critical roles in the modulation of lung tumor biology (Fig. 2A). These findings led us to further investigate the mechanisms underlying the regulation of EGFR expression in vivo, with a specific focus on epigenetic modifications. To this end, we conducted ChIP assays to examine the EGFR gene promoter and super-enhancer genetic sequences in the previously mentioned lung tumors. Our inquiry identified an enrichment of the EZH2 mark at EGFR genetic sequences, consistent with an increase in the repressive histone mark H3K27me3 under a reduced MEOX2 condition (shMEOX2). However, a decrease in EZH2 occupancy was observed under a reduced GLI-1 condition (shGLI-1), with no accompanying changes in H3K27me3. Such findings suggest how GLI-1 modulates the enrichment of EZH2 but does not influence H3K27me3 enrichment, in contrast to MEOX2 modulation capacity (Fig. 2B, C). Furthermore, shMEOX2 reduced the enrichment of CBP and histone mark H3K27Ac at EGFR genetic sequences, while shGLI-1 induced a similar decreased CBP and a trend toward reduced H3K27Ac levels. This indicates that both MEOX2 and GLI-1 are playing a role in modulating these epigenetic marks, although their effects on H3K27Ac enrichment levels may differ (Fig. 2D, E). Finally, under both shMEOX2/shGLI-1 conditions, we have detected little variations with an enrichment tendency of the SMARCB1 protein on EGFR genetic sequences (Fig. 2F). In addition, similar epigenetic variations were detected at the enhancer and promoter sequences of the GLI-1 oncogene, emphasizing the modulation capacity of the epigenetic marks mediated by both MEOX2 and GLI-1 transcription factors (Fig. S3). Overall, these epigenetic fluctuations suggest that MEOX2 and GLI-1 are functionally involved in transcriptional and epigenetic regulation of the EGFR oncogene expression in a Polycomb *versus* Trithorax dependent manner in solid lung tumors in vivo, highlighting an increased SMARCB1 occupancy on EGFR genetic sequences through the use of shMEOX2, suggesting the involvement of MEOX2 in SMARCB1 expression and its role in lung cancer progression.

### SMARCB1 suppresses lung tumor progression in vivo during EGFR-TKI-afatinib-based therapy

**Fig. 3:**
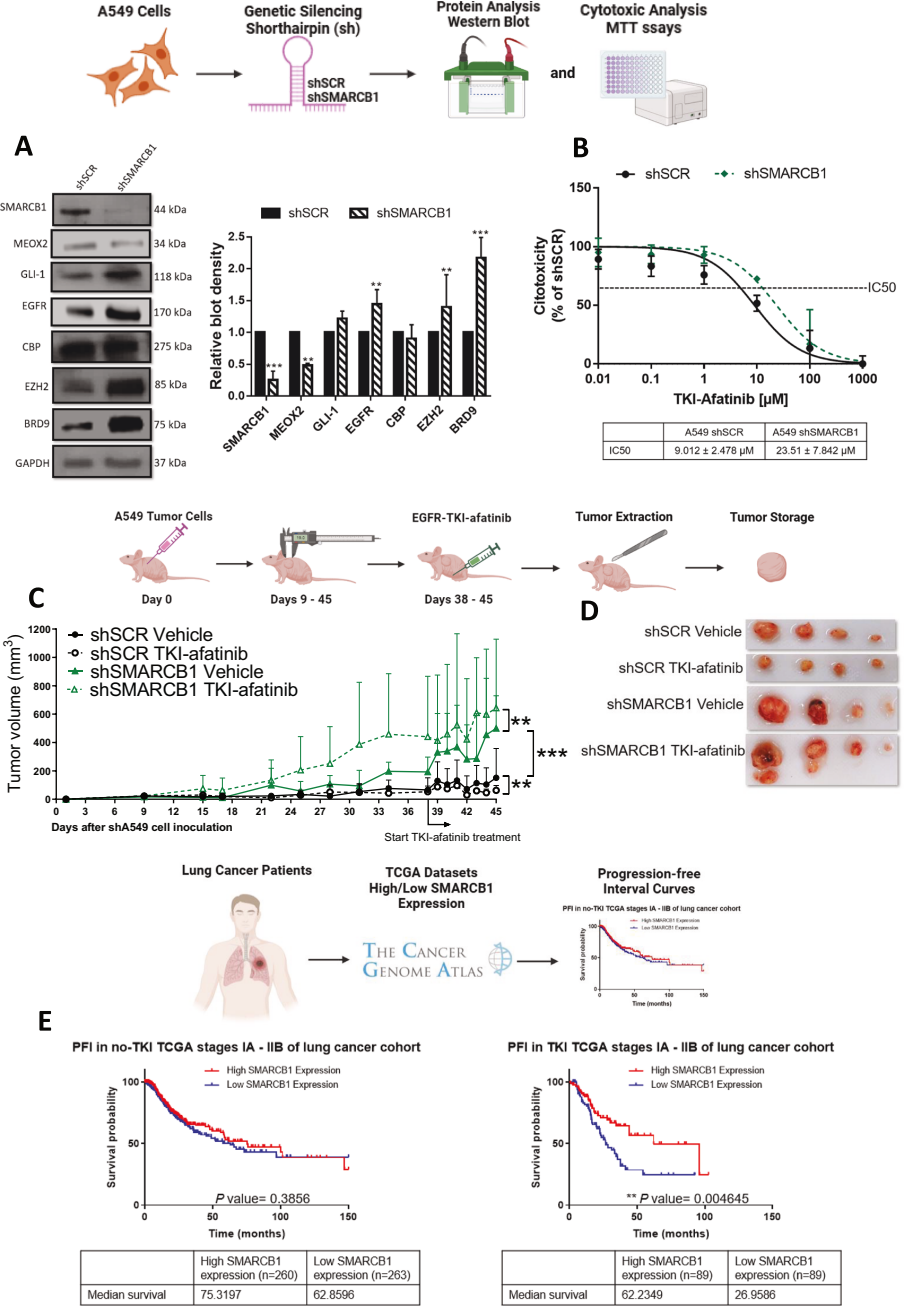
SMARCB1 gene silencing increases lung tumor cell progression in vivo and EGFR-TKI-afatinib resistance. **A** Cellular cultures of monolayer A549 lung cancer cells show that SMARCB1 was effectively silenced by shRNAs-SMARCB1, triggering a decrease in MEOX2 and an increase in GLI-1, EGFR, CBP, EZH2 and BRD9 protein abundance. Data represents the mean ± S.D. of three independent biological replicates. **B** Analysis of cytotoxicity against TKI-afatinib in negative control shSCR and shSMARCB1, identifying an inhibitory concentration 50 for each transfection condition. Data represents the mean ± S.D. of three independent biological replicates by triplicate. **C** Analysis of the tumor volume by each mouse experimental group, through in vivo tumor progression. Immuno-deficient nu-/nu- mice were organized into shSCR and shSMARCB1 experimental groups under treatment-free conditions “vehicle” and TKI-afatinib treatment (10 mg/kg). Tumor volume was measured twice weekly, represented as mean ± S.D. for 4 mice per group. Graph shows one experiment of two replicates. **D** Visual representation of tumor growth at the end of in vivo lung tumor cell progression, shSCR Vehicle (*n* = 4), shSCR TKI-afatinib (*n* = 4), shSMARCB1 Vehicle (*n* = 4) and shSMARCB1 TKI-afatinib (*n* = 4). **E** PFI analysis for SMARCB1-associated expression in earlier clinical stages (IA-IIB) of non-TKI-treated and TKI-treated lung cancer patients.

Based on this increased SMARCB1 occupancy caused by reduced MEOX2 expression and prior experimental reports of SMARCB1-dependent EGFR regulation in MRT cells [16], we analyzed SMARCB1 role in the regulation of EGFR expression. Therefore, by use of shSMARCB1, we downregulated SMARCB1 genetic expression (Fig. S2) and protein levels (Fig. 3A), identifying a significant decrease in MEOX2 expression and protein levels (Figs. S2, 3A) in A549 lung cancer cells. In contrast, the levels of GLI-1 and CBP remained unchanged, whereas protein levels of EGFR, EZH2, and BRD9 significantly increased (Fig. 3A). It is important to highlight the inclusion of BRD9 in our analysis, as the presence of BRD9 within the ncBAF assembly, lacking SMARCB1, may confer oncogenic properties. Our findings suggest that SMARCB1 and MEOX2 mutually regulate each other, with the loss of SMARCB1 promoting genetic modulation of EGFR, EZH2, and BRD9 (Fig. 3A). After determining a SMARCB1-Driven EGFR expression in lung cancer, we examined if this association had an impact on EGFR-TKI resistance. We specifically evaluated the EGFR-TKI-afatinib due to its irreversible binding capabilities, which offer a unique opportunity to examine the effects of sustained receptor inhibition. This approach may provide deeper insights into the dynamics of EGFR signaling and the role of SMARCB1 in modulating this cancer pathway. To assess this, we performed MTT in vitro assays and detected that SMARCB1 genetic silencing by shSMARCB1 promoted resistance to TKI-afatinib. This outcome suggests that the loss of SMARCB1 may enhance the activation of compensatory cancer signaling pathways, allowing A549 lung cancer cells to evade the inhibitory effects of this anti-cancer compound “afatinib” (Fig. 3B). This evidence let us confirm SMARCB1 as a tumor suppressor in lung cancer and prompted us to investigate lung cancer progression and response to the EGFR-TKI-afatinib at in vivo (nu-/nu-) mice model. Our analysis revealed a significant increase in tumor growth in the shSMARCB1 (vehicle experimental group) compared to the shSCR control, further supporting SMARCB1 tumor-suppressive role in lung cancer. Furthermore, the shSMARCB1 experimental group with TKI-afatinib-based therapy exhibited a significant resistance, evidenced by the increase in tumor growth, contrasting with the shSCR group under TKI-afatinib treatment, which showed a significant decrease in tumor volume. These results indicate that SMARCB1 not only functions as a tumor suppressor in lung cancer but also enhances tumor sensitivity to EGFR-TKI-afatinib treatment, in accordance with our in vivo lung cancer progression model (Fig. 3C, D). Additionally, to associate these findings with clinical outcomes of lung cancer patients, we examined a patient cohort from the TCGA Lung Cancer dataset at clinical early stages, indicating no significant difference in non-TKI-treated patients (75.31 vs. 62.85 months). Interestingly, despite the substantial increase in tumor size detected by our mice model following SMARCB1 genetic silencing, lower SMARCB1 expression did not appear to impact the PFI of non-TKI-treated patients. However, in EGFR-TKI-treated patients, high SMARCB1 expression significantly associated with extended PFI (62.23 vs. 26.95 months) (Fig. 3E). Furthermore, no significant associations were identified between SMARCB1 expression and PFI in advanced clinical-stage lung cancer patients (Fig. S1). Overall, these findings suggest an oncological potential of SMARCB1 as a prognosis biomarker for EGFR-TKI-based therapy responsiveness in lung cancer, uncovering its in vivo function as a tumor suppressor gene and a significant epigenetic actor in human lung cancer progression.

### SMARCB1-drives gene expression patterns and altered epigenetic marks H3K27me3/BRD9/MTA2 at EGFR/GLI-1 gene sequences in in vivo lung tumors

**Fig. 4:**
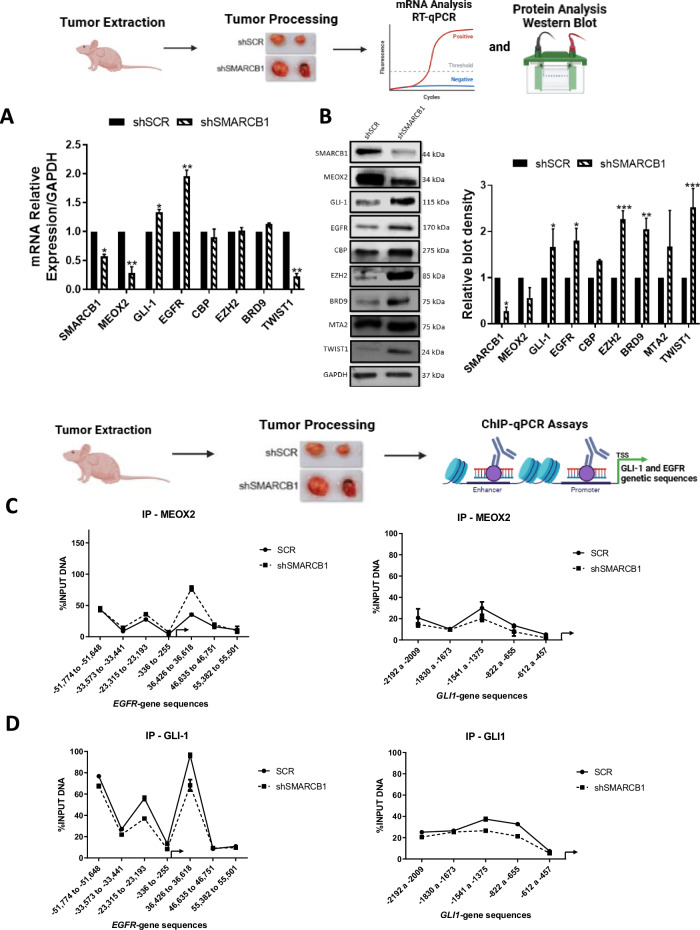

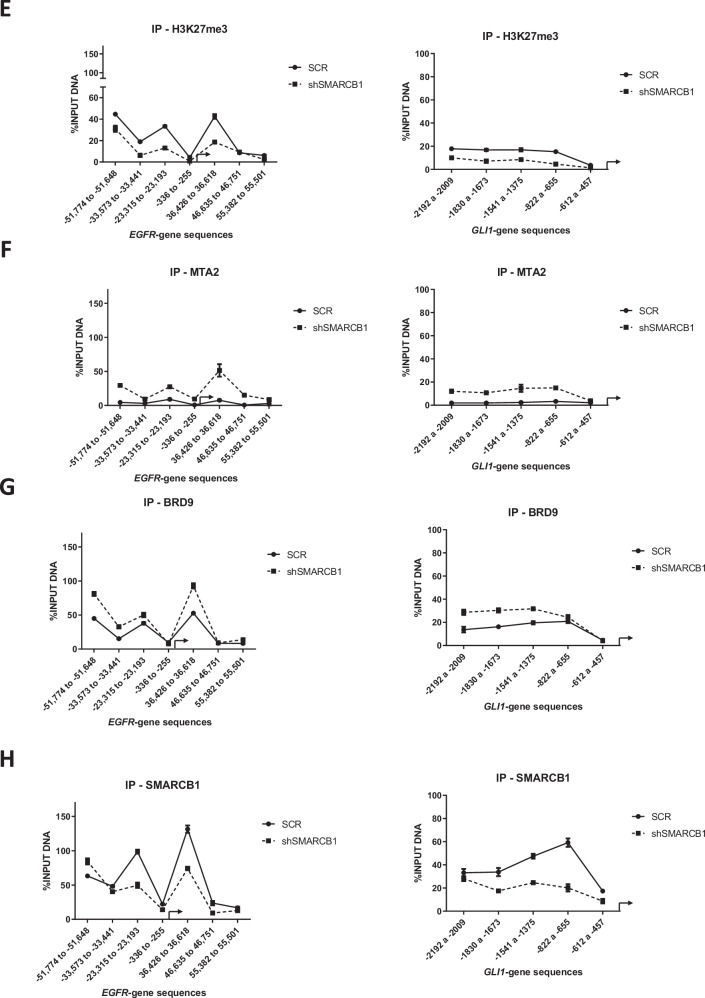
Genetic silencing of SMARCB1 increases EGFR and GLI-1 gene expression, altering epigenetic marks H3K27me3, BRD9, and MTA2. **A** In vivo lung tumors show that SMARCB1 was silenced by shRNAs, triggering changes on the expression of MEOX2, GLI-1, EGFR, CBP, EZH2, BRD9 and TWIST1. Data represents the mean ± S.D. of two independent biological replicates. **B** Western blot shown that genetic silencing of SMARCB1 decreases MEOX2 and increases GLI-1, EGFR, CBP, EZH2, BRD9, MTA2 and TWIST1 protein abundance. GAPDH was used as loading control. Data represents the mean ± S.D. of two independent biological replicates. **C** The occupation of MEOX2, **D** GLI-1, **E** H3K27me3, **F** MTA2, **G** BRD9 and **H** SMARCB1 were quantified by the effect of genetic silencing of SMARCB1 at super-enhancer and promoter sequences of the EGFR gene as well as the enhancer and promoter sequences of the GLI-1 gene, in lung tumors. Graph shows a single representative experiment of two biological replicates.

To understand the epigenetic impact of SMARCB1 genetic silencing in solid lung tumors, we conducted an analysis of mRNA and protein levels in in vivo lung tumors. By comparing untreated lung tumors under shSCR and shSMARCB1 conditions, derived from the lung tumor progression assays (Fig. 3D), we aimed to determine the impact of SMARCB1 suppression on oncogenes and chromatin remodelers. Our analysis revealed that SMARCB1 significant reduction led to altered gene expression and protein levels for MEOX2, CBP, EZH2, BRD9, MTA2, and TWIST1. Notably, this reduction showed a significant increase in both the gene expression and protein levels of both EGFR and GLI-1 oncogenes, highlighting their potential role in lung tumor progression under lower SMARCB1 expression (Fig. 4A, B). This finding prompted us to investigate whether their upregulation was driven by epigenetic changes derived from SMARCB1 protein reduction. To explore this, we conducted ChIP-qPCR assays on solid in vivo lung tumors, aiming to uncover specific chromatin alterations associated with EGFR and GLI-1 genetic expression. Our analysis showed that MEOX2 occupancy on both EGFR/GLI-1 genetic sequences was altered, while GLI-1 occupancy decreased under shSMARCB1 condition (Fig. 4C, D). In addition, H3K27me3 enrichment decreased in EGFR/GLI-1 sequences (Fig. 4E). Furthermore, an increase in the occupancy of the NuRD-complex member-MTA2, under shSMARCB1 condition was observed (Fig. 4F). Additionally, the occupancy of the ncBAF member BRD9 increases, contrasting with a decreased occupancy of SMARCB1 under shSMARCB1 condition on both EGFR/GLI-1 genetic sequences (Fig. 4G, H). These findings suggest SMARCB1 suppression enhances EGFR and GLI-1 genes expression through a H3K27me3 histone mark reduction, and a MTA2/BRD9 (NuRD/ncBAF) complex recruitment, hinting at their potential involvement in promoting genetic expression of both EGFR and GLI-1 oncogenes. In contrast, with little occupancy change on non-oncogene genetic sequences (Figs. S5 and S6).

### SMARCB1 protein-protein interaction with BRD9, GLI-1 and MEOX2, as potential epigenetic modulation in the therapy of human lung cancer cells

**Fig. 5:**
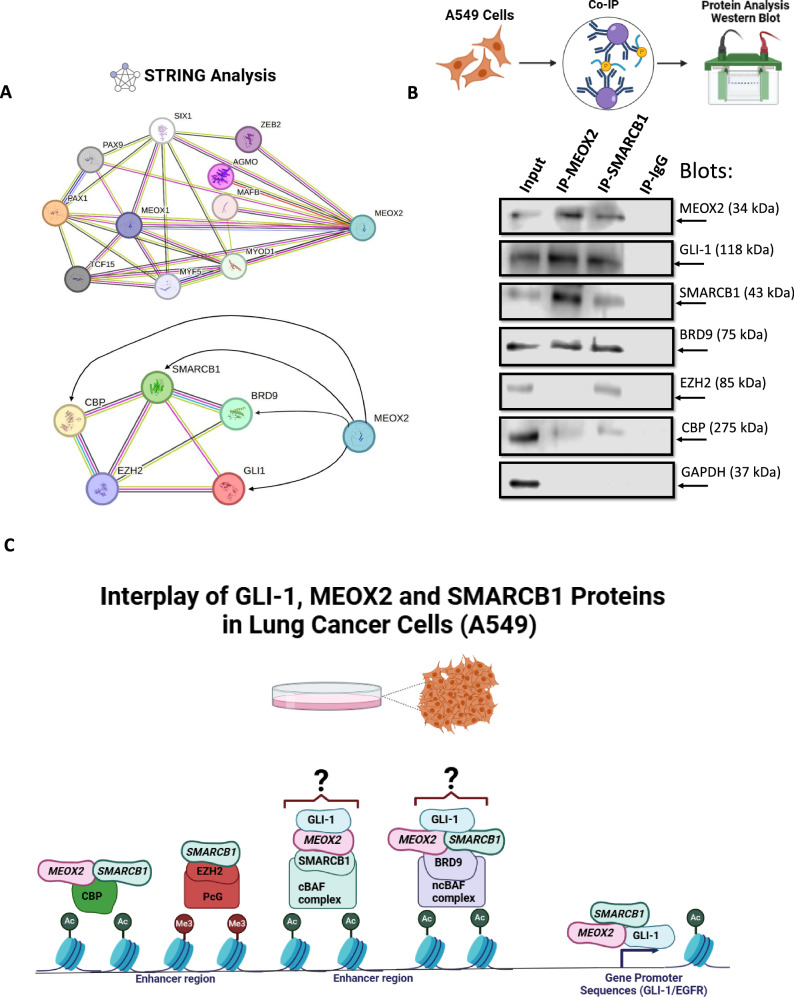
Interaction of MEOX2 and SMARCB1 with putative interacting proteins. **A** Predicted protein-protein interactions of MEOX2 with its reported interacting partners, as well as with GLI-1, SMARCB1, BRD9, EZH2, and CBP, based on analysis conducted using the STRING database. Interaction confidence was set to 0.400, and associations are visualized with corresponding network connections. **B** Co-IP assays showing the interactions between MEOX2 and SMARCB1 with GLI-1, EZH2, CBP and BRD9 in lung cancer cells (A549). GAPDH was used as a negative control. Data represents one biological replicate. **C** Model depicting the protein-protein interactions of GLI-1, MEOX2 and SMARCB1 alongside the hypothetical mechanisms governing the transcriptional regulation of EGFR/GLI-1 genes in lung cancer cells.

Upon assessing the occupancy changes in EGFR/GLI-1 genetic sequences induced by both MEOX2 and SMARCB1, we decided to explore the potential protein-protein interactions of MEOX2 and SMARCB1 using the STRING database. Initially, we documented previously reported interactions involving MEOX2, such as those with PAX1, PAX9, MEOX1, SIX1, and ZEB2 (Fig. 5A). Notably, while interactions among SMARCB1, GLI-1, EZH2, CBP, and BRD9 have been reported, no prior studies have indicated interactions between MEOX2 with these proteins (Fig. 5A). Based on this, we conducted Co-IP assays using A549 lung cancer cells, which revealed that SMARCB1 interacts with MEOX2, GLI-1, and BRD9, with a potential weak interaction with CBP and EZH2. In contrast, MEOX2 was found to interact with GLI-1, SMARCB1, and BRD9, but showed a slight interaction with CBP, however, no interaction with EZH2 was detected (Fig. 5B). These findings suggest, for the first time, that MEOX2 and GLI-1 interact with Trithorax complex members such as CBP, SMARCB1, and BRD9, underscoring a novel potential role for MEOX2 and GLI-1 in lung cancer biology. Through these protein-protein interactions, MEOX2 and GLI-1 may facilitate the modulation of epigenetic modifications, such as histone acetylation and methylation, on their oncological targets. Additionally, SMARCB1 interactions with CBP, EZH2, and BRD9 may impact transcriptional and epigenetic regulation of EGFR and GLI-1 oncogenes. In conclusion, our results suggest novel molecular mechanisms whereby MEOX2 and GLI-1 may modulate functional dynamics between cBAF (SMARCB1) and ncBAF (BRD9) complexes, contributing to lung cancer progression (Fig. 5C).

## Discussion

Genetic silencing of MEOX2 and GLI-1 in NSCLC cells revealed significant changes in EGFR, CBP and SMARCB1 protein levels, indicating the potential regulatory roles of MEOX2/GLI-1 in accordance with preceding studies [8]. Nevertheless, this is the first report suggesting that MEOX2/GLI-1 may modulate CBP protein levels.

In vivo tumor xenograft assays using nu-/nu- mice showed that shMEOX2 significantly decreased tumor size, indicating MEOX2 promotes lung tumor growth. These findings are consistent with prior reports associating MEOX2 with proliferation and tumor promotion, suggesting that MEOX2 may facilitate tumor growth through its mechanistic involvement with key hallmarks of cancer [4, 8, 33]. Conversely, the shGLI-1 group showed increased tumor size, which contradicts previous studies where GLI-1 has been shown to promote proliferation and tumor growth in lung cancer [5, 7, 34]. To our knowledge, this is the first report indicating a suppressive effect of GLI-1 on lung cancer tumor growth. Furthermore, treatment with EGFR-TKI erlotinib significantly decreased tumor size in the shMEOX2 group, indicating a potential interaction between MEOX2 and EGFR signaling. These observations align with previous reports implicating MEOX2 in response to EGFR-TKIs [8]. The shGLI-1 group showed no significant tumor size reduction, indicating that GLI-1 may not significantly influence EGFR-TKI erlotinib response, contrary to previous research suggesting GLI-1 suppression enhances sensitivity to erlotinib [35], suggesting that GLI-1 may not directly influence EGFR signaling in lung cancer cells. These findings highlight the potential of MEOX2 expression levels in predicting the response to EGFR-targeted therapy in lung cancer patients.

To assess the clinical significance of MEOX2 and GLI-1 expression in lung cancer, we analyzed the TCGA Lung Cancer dataset, focusing on PFI curves in early-stage patients. Higher MEOX2 expression was linked to shorter median PFIs, both in untreated and TKI-treated patients. This supports previous research showing that lung cancer patients with lower MEOX2 expression and EGFR mutations had better overall survival with combined cisplatinum and TKI therapy [4]. Conversely, TKI-treated patients with higher GLI-1 expression had longer median PFIs than those with lower expression levels. Our findings contrast with previous studies in lung cancer patients, where those with low GLI-1 expression showed significantly better progression-free survival with EGFR-TKI erlotinib treatment compared to those with high GLI-1 expression [35]. These findings indicate divergent roles for MEOX2 and GLI-1 in lung cancer progression, suggesting MEOX2 expression levels could predict clinical outcomes in early-stage patients.

To elucidate the mechanisms regulating EGFR expression, we investigated the role of epigenetic modifications. In vivo solid lung tumor samples subjected to shMEOX2 and shGLI-1 conditions, we observed differential enrichment of epigenetic regulators at EGFR-associated sequences. Specifically, shMEOX2 treatment resulted in increased enrichment of EZH2 at EGFR loci, which was consistent with elevated levels of the repressive histone mark H3K27me3. This suggests that in the absence of MEOX2, EGFR repression is modulated through EZH2, subsequently decreasing EGFR expression in lung tumors. Consistent with our findings, it has been reported that HLX1, a homeobox protein, regulates the enrichment of Polycomb proteins and H3K27me3 at the INK4a/ARF locus in non-malignant lung cell lines [36]. This suggests a potential role for homeobox proteins, such as MEOX2, in the modulation of repressive epigenetic marks.

Conversely, shGLI-1 was associated with a reduction in EZH2 enrichment at EGFR sequences with no change in H3K27me3, indicating that GLI-1 may not impact repressive histone marks governing EGFR gene regulation. Accordingly, in medulloblastoma cells, the SHH protein has been shown to upregulate EZH2, indicating that the Sonic Hedgehog pathway plays a role in the modulation of Polycomb proteins [37]. In parallel, changes in CBP and H3K27Ac enrichment at the EGFR super-enhancer and promoter regions were observed under both shMEOX2 and shGLI-1 conditions. Reduced CBP and H3K27Ac levels under these conditions suggest that MEOX2 and GLI-1 may influence active histone modifications via CBP, potentially modulating EGFR gene expression. Our findings align with previous reports indicating that genetic silencing of MEOX2 and GLI-1 reduces H3K27Ac enrichment at the EGFR locus in NSCLC cells [8]. Notably, our study is the first to demonstrate this effect in solid in vivo lung tumors. Additionally, for the first time, our findings reveal that MEOX2 may modulate SMARCB1 enrichment, as evidenced by the notable increase in SMARCB1 at EGFR super-enhancer regions under shMEOX2 conditions. This novel role of MEOX2 in regulating SMARCB1 recruitment suggests its influence on the epigenetic landscape of the EGFR gene, underscoring the involvement of MEOX2 in modulating both repressive and activating chromatin states in lung tumors.

Further investigation of SMARCB1 revealed its potential tumor suppressor role in lung cancer. Genetic silencing in A549 cells significantly increased EZH2, EGFR, and BRD9 protein levels, consistent with observations in MRT [16, 38, 39]. The association between SMARCB1 and EGFR significantly influenced lung cancer cell sensitivity to EGFR-TKI-afatinib, evident in both in vitro and in vivo settings. Tumor xenograft assays demonstrated resistance to EGFR-TKI-afatinib in the shSMARCB1 group, with a notable increase in tumor size upon SMARCB1 silencing. These findings underscore the tumor suppressor role of SMARCB1 in lung cancer, consistent with prior studies across various cancer types [12, 40–42]. Furthermore, it has been mentioned that mSWI/SNF subunits play a significant role in the development of several human lung diseases, including lung malignant diseases [23]. Moreover, in MRT cells, EGFR-TKIs such as Lapatinib or Gefitinib reduce cell proliferation and hinder tumor activity in SMARCB1-deficient cells and tumors [16, 43]. These findings contrast with our results using EGFR-TKI afatinib. Thus, this study is the first to show that SMARCB1-deficient tumors resist EGFR-TKIs in cancer.

Clinical analysis of SMARCB1 in a TCGA lung cancer dataset revealed that low SMARCB1 expression did not affect PFI in non-TKI-treated patients. In contrast, our in vivo mouse model demonstrated a substantial increase in tumor size following genetic silencing of SMARCB1. This discrepancy may be attributed to the fact that PFI reflects more than just tumor growth, it also encompasses processes such as metastasis and immune system interactions, which are influenced by factors such as the tumor microenvironment and patient-specific variability [44, 45]. These elements, which are not fully captured in mouse models, may explain the observed difference between clinical and preclinical findings.

Nevertheless, higher SMARCB1 expression correlated with significant extended PFIs in TKI-treated early-stage patients compared to lower levels, consistent with studies linking mSWI/SNF subunit alterations to reduced progression-free survival in EGFR-TKI therapy for lung cancer [46]. These results highlight the impact of SMARCB1 on EGFR expression and its potential role in determining response to EGFR-TKIs in lung cancer, offering new insights into its clinical relevance.

The association between SMARCB1 and EGFR was confirmed in solid in vivo lung tumors, where SMARCB1 suppression increased EGFR mRNA and protein levels, aligning with previous reports on SMARCB1-dependent EGFR activation in MRT cells [16]. Additionally, the significant increase in GLI-1 expression and protein levels observed in in vivo lung tumors under the genetic silencing of SMARCB1 aligns with findings in MRT in which loss of SMARCB1 leads to aberrant activation of GLI-1 [47].

To explore the molecular mechanisms of EGFR and GLI-1 regulation by SMARCB1 in lung tumors, ChIP assays were performed, revealing changes in GLI-1/MEOX2 occupancy under shSMARCB1 conditions, indicating potential modulation by SMARCB1, accordingly, it has been reported that SMARCB1 interacts with GLI-1 in MRT [47], but this is to our knowledge the first report of SMARCB1 modulating the occupancy of MEOX2. Additionally, increased EZH2 occupancy, despite reduced H3K27me3 levels, highlights the complex regulatory mechanisms governing EGFR/GLI-1 expression, involving multiple epigenetic factors. Consistent with previous studies, our findings confirm the antagonistic relationship between SMARCB1 and EZH2 in gene regulation [38, 48]. Notably, it has been documented that the deficiency of SMARCB1 leads to a global reduction in H3K27me3 [49], which is in line with our current observations. These results suggest EZH2 may have additional non-canonical roles in gene regulation, independent of its methyltransferase activity.

The increase in MTA2 occupancy suggests the NuRD complex may activate EGFR/GLI-1 expression under shSMARCB1 conditions, consistent with reports of mSWI/SNF and NuRD complexes antagonistically regulating DNA accessibility [50, 51]. Moreover, it has been reported that NuRD may activate gene transcription [52], interestingly, in embryonic stem cells lacking NuRD, EGFR expression was significantly downregulated [53]. No change in TWIST1 occupancy suggests MTA2 guides NuRD to EGFR/GLI-1, supported by reports indicating MTA proteins as core NuRD scaffolds recruiting multiple proteins that interact with histones, DNA, and nucleosomes [54]. Reduced SMARCB1 occupancy and increased BRD9 under shSMARCB1 suggest ncBAF complex assembly at EGFR/GLI-1, these findings align with previous reports that have highlighted the assembly of ncBAF in response to SMARCB1 deficiency [25, 27, 39, 55]. Additionally, there is evidence suggesting that BRD9 plays a role in activating oncogenes in leukemias [24, 26] and lung cancer [30]. Hence, it is plausible to propose that BRD9 might be involved in the activation of EGFR/GLI-1 in the absence of SMARCB1. Furthermore, these findings allow us to discuss that the inhibition of BRD9 may improve the therapeutical responses in lung cancer patients. This possibility is supported by reports indicating that selective chemical probes targeting BRD9 (inhibitors) when applied in NSCLC cell lines, significantly increased sensitivity to EGFR-TKIs [56].

Moreover, to our knowledge, this is the first time that the protein-protein interaction of MEOX2, with GLI-1, SMARCB1, BRD9 and CBP is ever shown, but it has been reported that homeobox proteins (HOXB13) interact with subunits of mSWI/SNF (SMARCC1) in prostate cancer [57], in agreement with our results in lung cancer. Furthermore, in rhabdomyosarcoma, evidence indicates a protein-protein interaction between members of mSWI/SNF (SMARCA2) and GLI-1, consistent with our findings in lung cancer, where SMARCB1 interacts with GLI-1 [58]. In contrast, investigations in mouse embryonic stem cells show an interaction between SMARCB1 and mSWI/SNF subunits ARID1A, SMARCA4, and SMARCC1, with no interaction observed with BRD9 [55], which contradicts our preliminary results in lung cancer cells, where we have suggested that SMARCB1 may interact with BRD9. Moreover, in MRT, evidence indicates that SMARCB1 interacts with CBP, aligning with our observations in lung cancer [59]. Additionally, protein-protein interactions between EZH2 and SMARCB1 have been identified in eosinophilic leukemia cells, corroborating our findings in lung cancer [60].

In conclusion, this study reveals the critical roles of MEOX2, GLI-1, and SMARCB1 as tumor biomarkers in human lung cancer progression. It identifies MEOX2 as a potential early-stage prognostic biomarker that may drive EGFR-TKI resistance, potentially through modulation of the mSWI/SNF complex. Additionally, SMARCB1 functions as a tumor suppressor by regulating EGFR gene expression and modulating cellular response to EGFR-TKIs in human lung cancer. Furthermore, this study suggests that epigenetic regulators, including the NuRD (MTA2) and ncBAF (BRD9) complexes, contribute to EGFR and GLI-1 expression and interact with MEOX2 in lung cancer cells. Together, these findings enhance our understanding of the molecular mechanisms underlying human lung cancer and underscore the potential of developing novel inhibitors targeting MEOX2, GLI-1, and ncBAF complex subunits, as previously proposed in lung disease studies [23], while also emphasizing the potential therapeutic value of reinducing SMARCB1 expression in human lung malignant diseases.

## Supplementary information

Supplementary Figures

Supplementary Tables

## Data Availability

The data that support the findings of our study are available from the corresponding author upon reasonable request.
