# Supplementary material for: SMARCB1-driven EGFR-GLI1 epigenetic alterations in lung cancer progression and therapy are differentially modulated by MEOX2 and GLI-1
Source: Cancer Gene Ther. 2025 Feb 19;32(3):327–42. doi: 10.1038/s41417-025-00873-0 (PMC11946902; doi:10.1038/s41417-025-00873-0)
Supplement: Supplementary file 1 — Supplementary Figures [file 41417_2025_873_MOESM1_ESM.pdf]

A

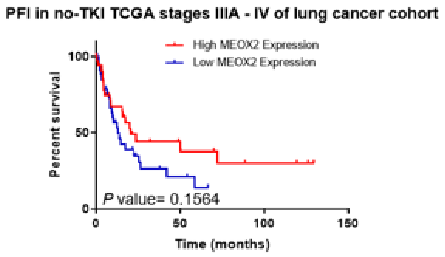

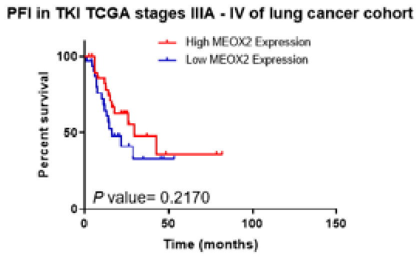

	High MEOX2 expression (n=35)	Low MEOX2 expression (n=35)
Median survival	20.5477	12.8875

	High MEOX2 expression (n=30)	Low MEOX2 expression (n=32)
Median survival	29.7531	16.3067

B

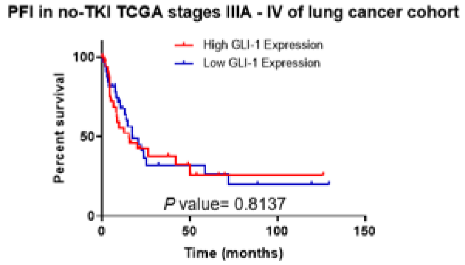

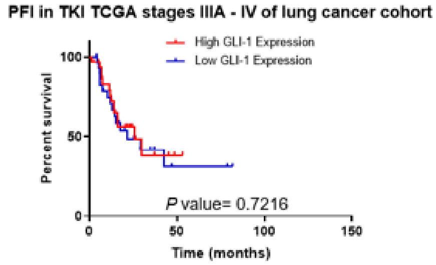

	High GLI-1 expression (n=35)	Low GLI-1 expression (n=35)
Median survival	15.3861	17.4573

	High GLI-1 expression (n=31)	Low GLI-1 expression (n=31)
Median survival	26.1367	21.5669

C

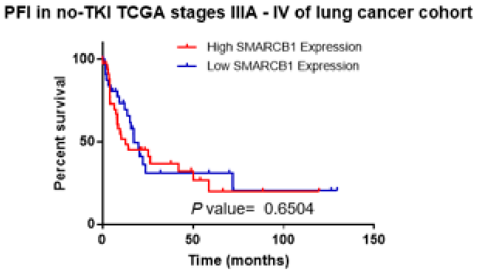

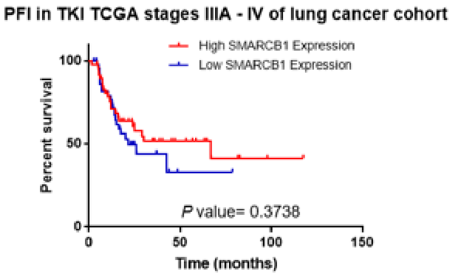

	High SMARCB1 expression (n=36)	Low SMARCB1 expression (n=34)
Median survival	12.8875	17.4573

	High SMARCB1 expression (n=49)	Low SMARCB1 expression (n=47)
Median survival	66.8376	21.5669

Fig. S1. Progression-free interval (PFI) analysis of MEOX2, GLI-1, and SMARCB1 expression in advanced clinical stage lung cancer patients (Stages IIIA-IV)*.* (A) PFI analysis for MEOX2-associated expression in late clinical stages of non-TKI treated and TKI treated lung cancer patients, **(B)** PFI analysis for GLI-1-associated expression in late clinical stages of non-TKI treated and TKI treated lung cancer patients. **(C)** PFI analysis for SMARCB1-associated expression in late clinical stages of non-TKI treated and TKI treated lung cancer patients.

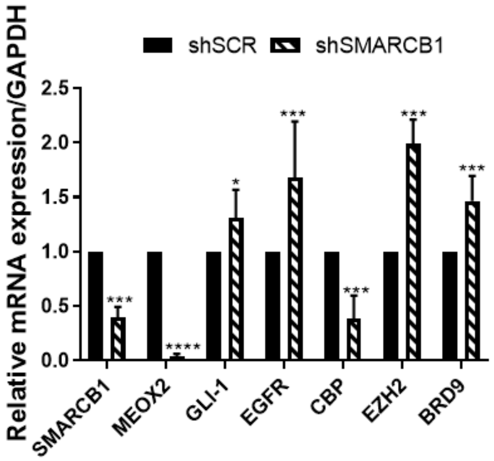

Fig. S2. Effect of the genetic silencing of SMARCB1 in mRNA expression in A549 monolayer lung cancer cells. A549 cells show that SMARCB1 was silenced by shRNAs, triggering a decrease in MEOX2 and CBP, and an increase in GLI-1, EGFR, EZH2 and BRD9 mRNA expression. Data represents the mean ± S.D. of three independent biological replicates.

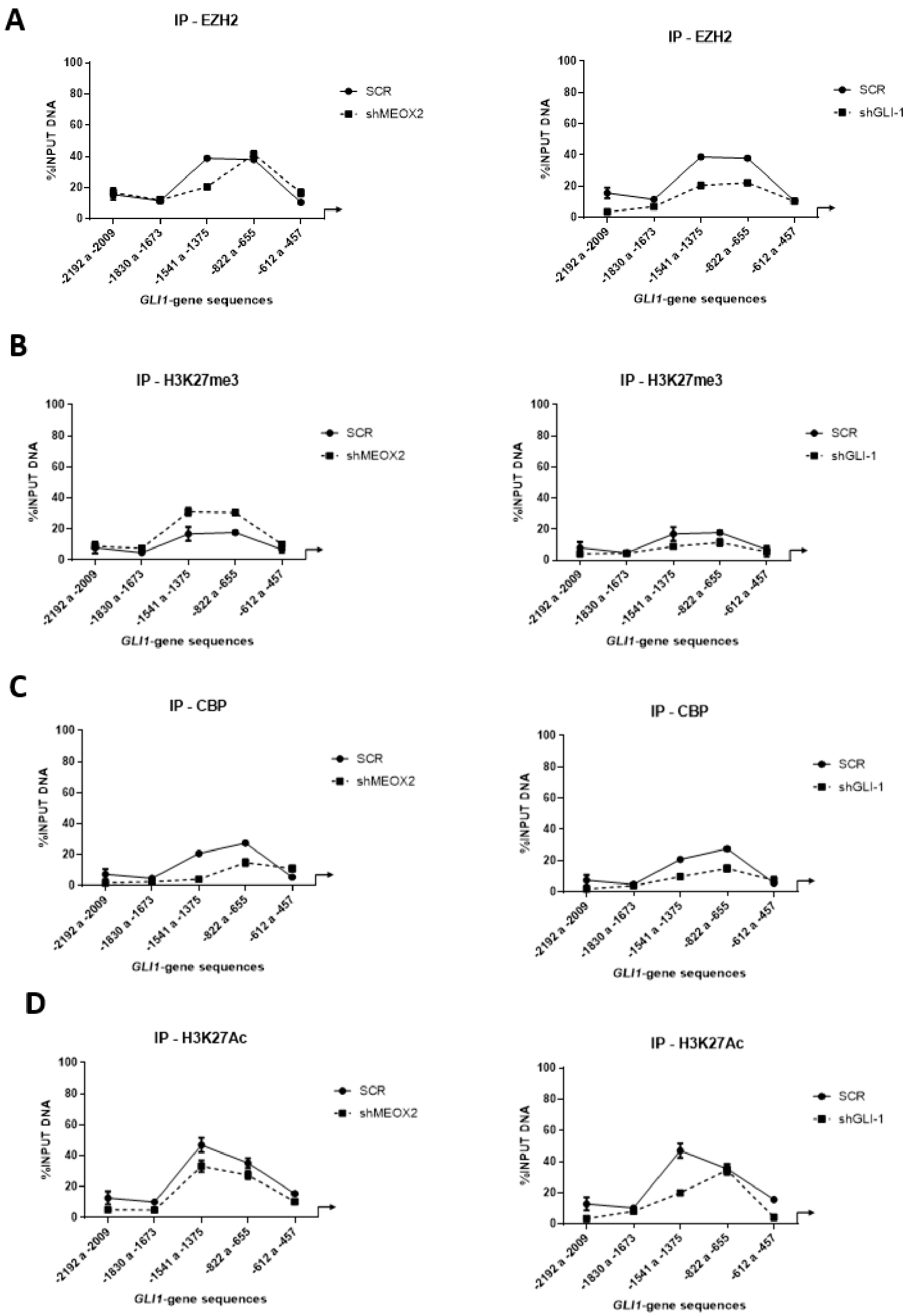
A
IP - EZH2
SCR
shMEOX2
%INPUT DNA
GLI1-gene sequences
-2192 a -2009
-1830 a -1673
-1541 a -1375
-822 a -655
-612 a -457
IP - EZH2
SCR
shGLI-1
B
IP - H3K27me3
SCR
shMEOX2
IP - H3K27me3
SCR
shGLI-1
C
IP - CBP
SCR
shMEOX2
IP - CBP
SCR
shGLI-1
D
IP - H3K27Ac
SCR
shMEOX2
IP - H3K27Ac
SCR
shGLI-1

E

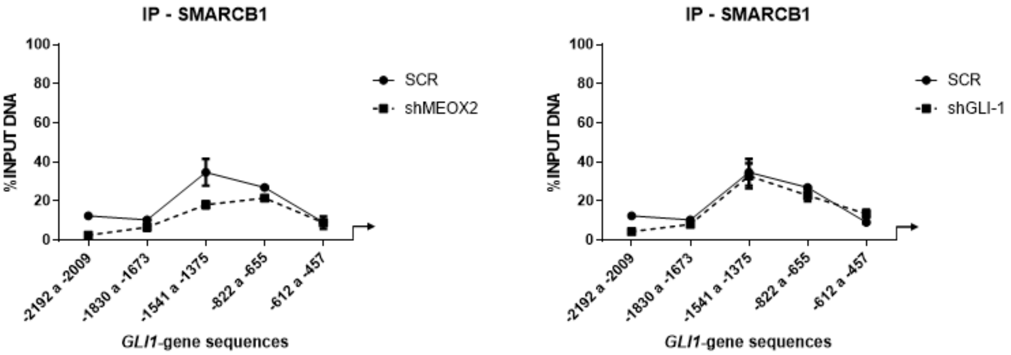

Fig. S3. Genetic silencing of MEOX2/GLI-1 alters *GLI-1* gene expression, modifying epigenetic marks EZH2/H3K27me3, and CBP/H3K27Ac at GLI1-gene promoter in solid *ex vivo* lung tumors*.* (A) The occupation of EZH2**(B)** H3K27me3 **(C)** CBP **(D)** H3K27Ac and **(E)** SMARCB1 were quantified by the effect of genetic silencing of MEOX2/GLI-1 at promoter and enhancer sequences of the *GLI-1* gene, in lung tumors.

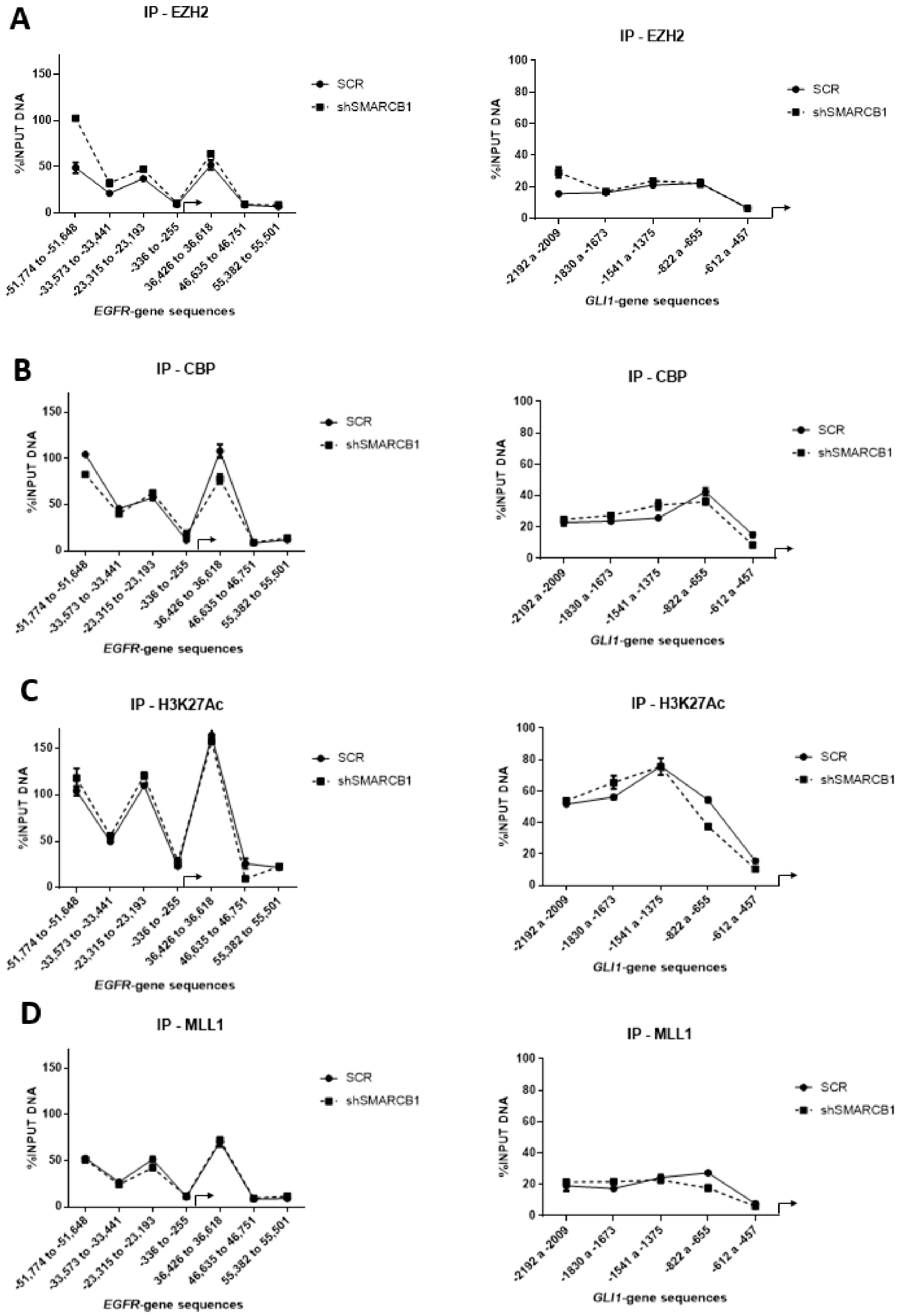
A
IP - EZH2
%INPUT DNA
SCR
shSMARCB1
-51,774 to -51,648
-33,573 to -33,441
-23,315 to -23,193
-336 to -255
36,426 to 36,618
46,635 to 46,751
55,382 to 55,501
EGFR-gene sequences
IP - EZH2
%INPUT DNA
SCR
shSMARCB1
-2192 a -2009
-1830 a -1673
-1541 a -1375
-822 a -655
-612 a -457
GLI1-gene sequences
B
IP - CBP
%INPUT DNA
SCR
shSMARCB1
EGFR-gene sequences
IP - CBP
%INPUT DNA
SCR
shSMARCB1
GLI1-gene sequences
C
IP - H3K27Ac
%INPUT DNA
SCR
shSMARCB1
EGFR-gene sequences
IP - H3K27Ac
%INPUT DNA
SCR
shSMARCB1
GLI1-gene sequences
D
IP - MLL1
%INPUT DNA
SCR
shSMARCB1
EGFR-gene sequences
IP - MLL1
%INPUT DNA
SCR
shSMARCB1
GLI1-gene sequences

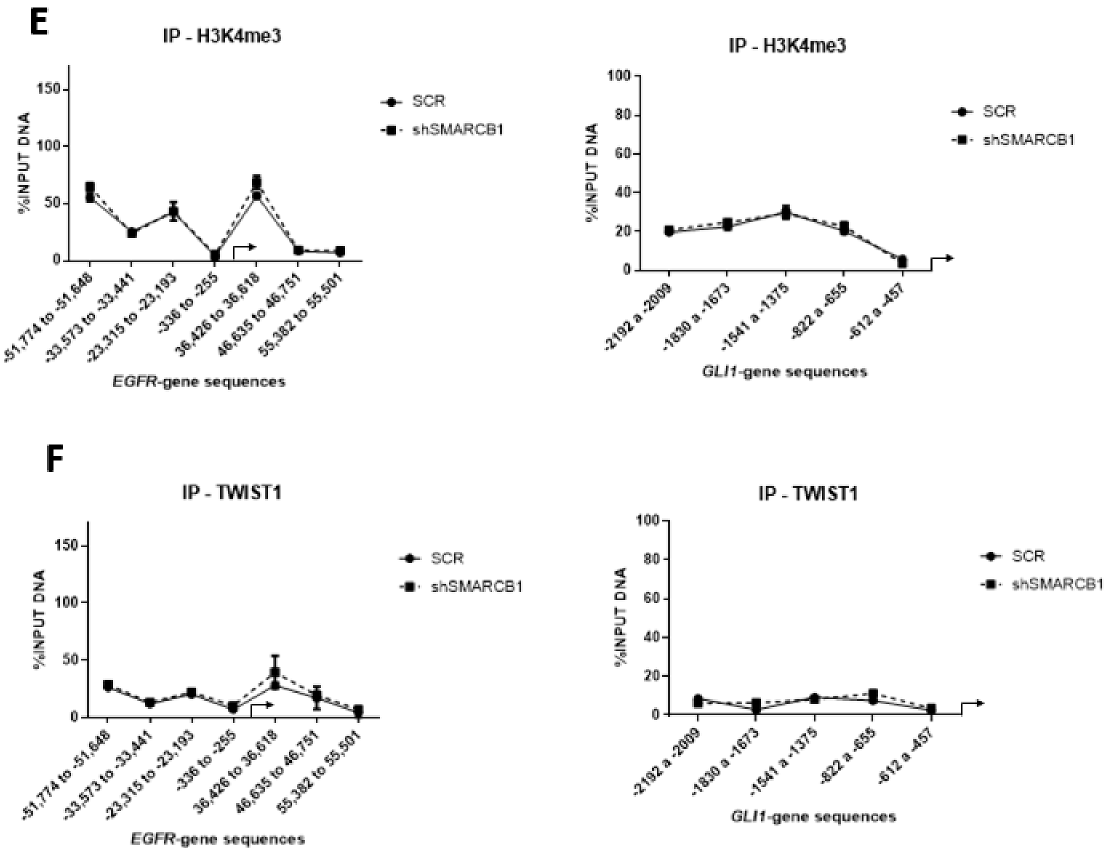

Fig. S4. Effect of the genetic silencing of SMARCB1 at *EGFR* and *GLI-1* gene sequences (A) The occupation of EZH2, **(B)** CBP, **(C)** H3K27Ac, **(D)** MLL1, **(E)** H3K4me3 and **(F)** TWIST1 were quantified by the effect of genetic silencing of SMARCB1 at super-enhancer and promoter sequences of the *EGFR* gene as well as the enhancer and promoter sequences of the *GLI-1* gene, in lung tumors.

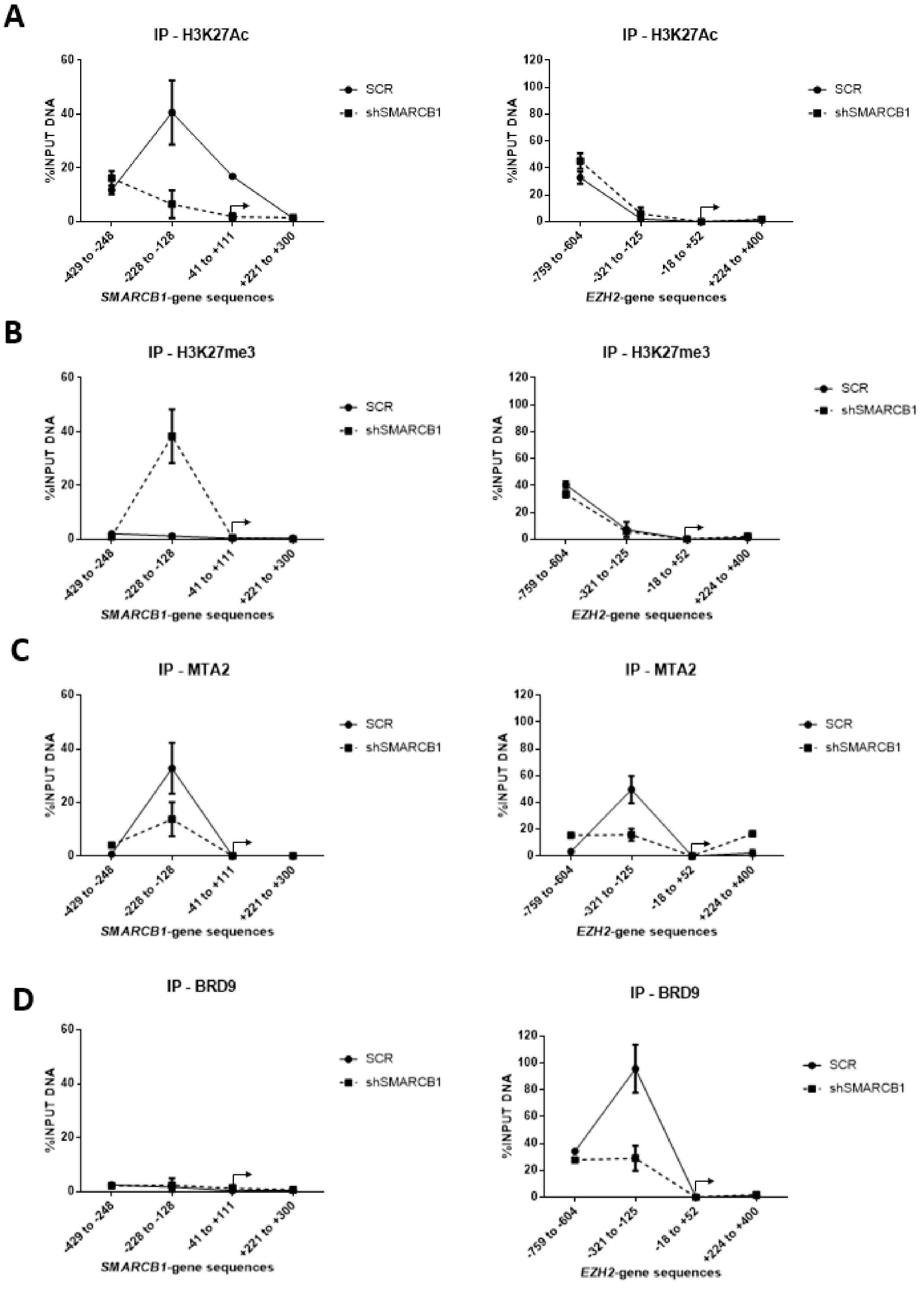
A
IP - H3K27Ac
%INPUT DNA
SCR
shSMARCB1
-429 to -248
-228 to -128
-41 to +111
+221 to +300
SMARCB1-gene sequences
IP - H3K27Ac
-759 to -604
-321 to -125
-18 to +52
+224 to +400
EZH2-gene sequences
B
IP - H3K27me3
IP - H3K27me3
C
IP - MTA2
IP - MTA2
D
IP - BRD9
IP - BRD9

Fig. S5. Genetic silencing of SMARCB1, modifies epigenetic marks at *SMARCB1*-gene and *EZH2*-gene sequences in solid *ex vivo* lung tumors. (A) The occupation of H3K27Ac, **(B)** H3K27me3, **(C)** BRD9 **(D)** and MTA2 were quantified by the effect of genetic silencing of SMARCB1 at promoter and enhancer sequences of the *SMARCB1*-gene and *EZH2*-gene, in lung tumors.

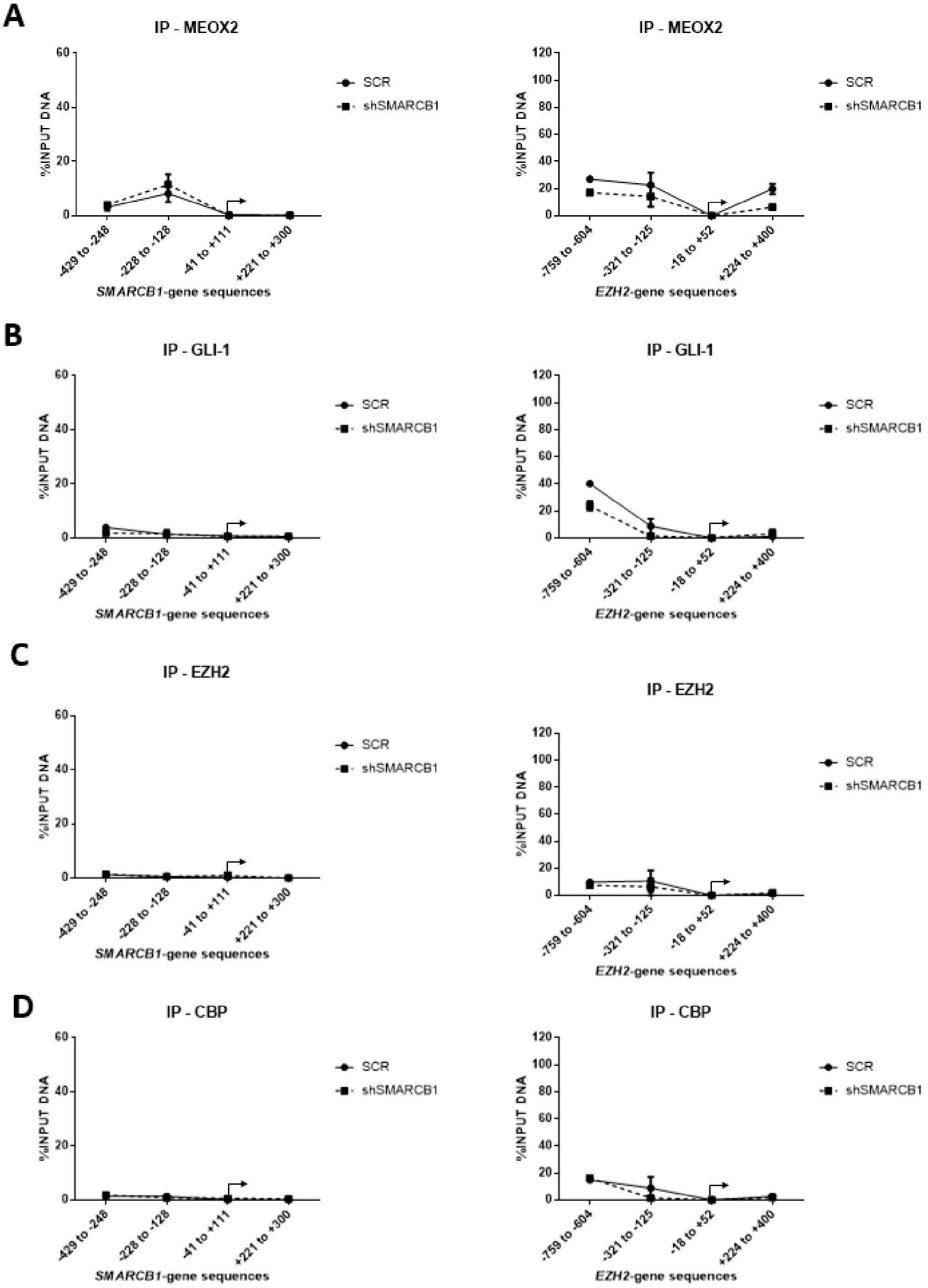
A
IP - MEOX2
%INPUT DNA
SCR
shSMARCB1
-429 to -248
-228 to -128
-41 to +111
+221 to +300
SMARCB1-gene sequences
IP - MEOX2
%INPUT DNA
SCR
shSMARCB1
-759 to -604
-321 to -125
-18 to +52
+224 to +400
EZH2-gene sequences
B
IP - GLI-1
%INPUT DNA
SCR
shSMARCB1
-429 to -248
-228 to -128
-41 to +111
+221 to +300
SMARCB1-gene sequences
IP - GLI-1
%INPUT DNA
SCR
shSMARCB1
-759 to -604
-321 to -125
-18 to +52
+224 to +400
EZH2-gene sequences
C
IP - EZH2
%INPUT DNA
SCR
shSMARCB1
-429 to -248
-228 to -128
-41 to +111
+221 to +300
SMARCB1-gene sequences
IP - EZH2
%INPUT DNA
SCR
shSMARCB1
-759 to -604
-321 to -125
-18 to +52
+224 to +400
EZH2-gene sequences
D
IP - CBP
%INPUT DNA
SCR
shSMARCB1
-429 to -248
-228 to -128
-41 to +111
+221 to +300
SMARCB1-gene sequences
IP - CBP
%INPUT DNA
SCR
shSMARCB1
-759 to -604
-321 to -125
-18 to +52
+224 to +400
EZH2-gene sequences

E

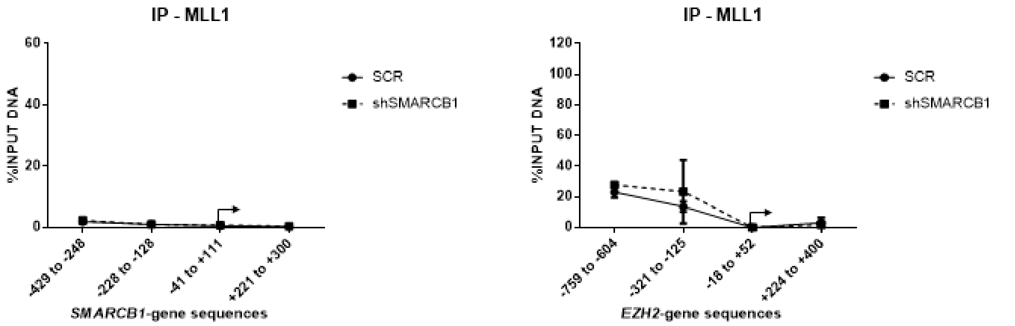
IP - MLL1
%INPUT DNA
-429 to -248
-228 to -128
-41 to +111
+221 to +300
SMARCB1-gene sequences
SCR
shSMARCB1
IP - MLL1
%INPUT DNA
-759 to -604
-321 to -125
-18 to +52
+224 to +400
EZH2-gene sequences
SCR
shSMARCB1

F

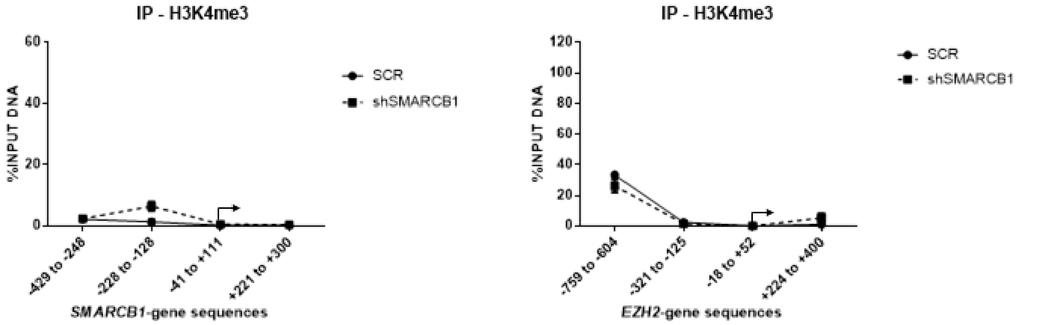
IP - H3K4me3
%INPUT DNA
-429 to -248
-228 to -128
-41 to +111
+221 to +300
SMARCB1-gene sequences
SCR
shSMARCB1
IP - H3K4me3
%INPUT DNA
-759 to -604
-321 to -125
-18 to +52
+224 to +400
EZH2-gene sequences
SCR
shSMARCB1

G

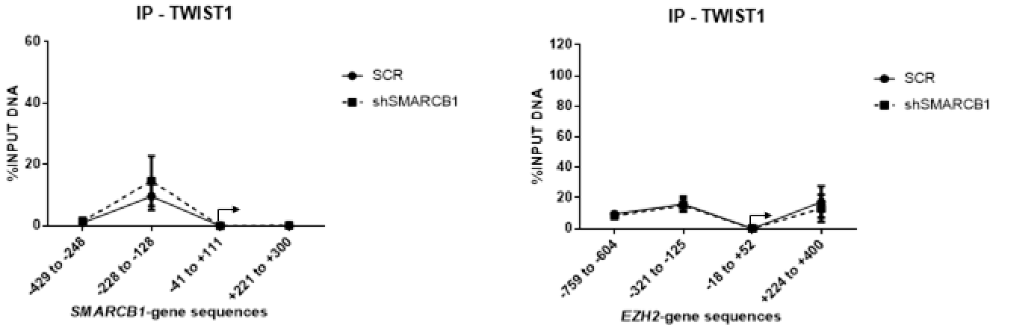
IP - TWIST1
%INPUT DNA
-429 to -248
-228 to -128
-41 to +111
+221 to +300
SMARCB1-gene sequences
SCR
shSMARCB1
IP - TWIST1
%INPUT DNA
-759 to -604
-321 to -125
-18 to +52
+224 to +400
EZH2-gene sequences
SCR
shSMARCB1

H

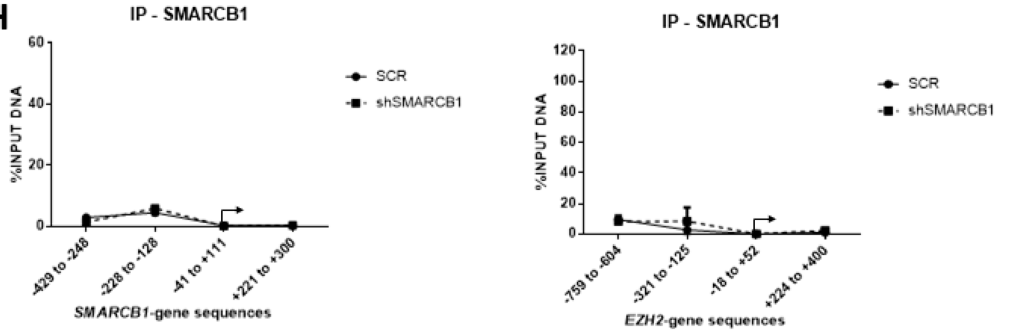
IP - SMARCB1
%INPUT DNA
-429 to -248
-228 to -128
-41 to +111
+221 to +300
SMARCB1-gene sequences
SCR
shSMARCB1
IP - SMARCB1
%INPUT DNA
-759 to -604
-321 to -125
-18 to +52
+224 to +400
EZH2-gene sequences
SCR
shSMARCB1

Fig. S6. Effect of the genetic silencing of SMARCB1 at *SMARCB1*-gene and *EZH2*-gene sequences in solid *ex vivo* lung tumors. (A) The occupation of MEOX2, **(B)** GLI-1, **(C)** EZH2, **(D)** CBP, **(E)** MLL1, **(F)** H3K4me3, **(G)** TWIST1, **(H)** and SMARCB1 were quantified by the effect of genetic silencing of SMARCB1 at promoter and enhancer sequences of the *SMARCB1*-gene and *EZH2*-gene, in lung tumors.
