# Supplementary material for: SMARCB1-driven EGFR-GLI1 epigenetic alterations in lung cancer progression and therapy are differentially modulated by MEOX2 and GLI-1
Source: Cancer Gene Ther. 2025 Feb 19;32(3):327–42. doi: 10.1038/s41417-025-00873-0 (PMC11946902; doi:10.1038/s41417-025-00873-0)
Supplement: Supplementary file 2 — Supplementary Tables [file 41417_2025_873_MOESM2_ESM.pdf]

Supplemental Table 1. Gene specific primers for mRNA analysis.

Gene	Primer (F=forward; R=reverse)	Product length (bp)
MEOX2	F: 5’-gtcagaagtcaacagcaaacccag-3<br>R: 5’-cacattcaccagttccttttcccgagcc-3’	247
GLI-1	F: 5’-ccaggaatttgactcccaag-3’<br>R: 5’-gggtttgaagggcctcag-3’	124
SMARCB1	F: 5’-acagtggagattgccatccg-3’<br>R: 5’-acgcctcatccgcctcgtgt-3’	129
EGFR	F: 5’-gcgtctcttgccggaatgt-3’<br>R: 5’-ggctcaccctccagaaggtt-3’	70
CBP	F: 5’-aaagcctgccaagccatcct-3’<br>R: 5’-ccctgtgccaacagaaccaa-3’	72
EZH2	F: 5’-gtacacggggatagagaatgtgg-3’<br>R: 5’-ggtgggcggctttctttatca-3’	176
BRD9	F: 5’-ggcaaggccgaaaatgagagc-3’<br>R: 5’-gcatccgtgacaggaaaagca-3’	110
TWIST1	F: 5’-gagtccgcagtcttacgag-3’<br>R: 5’-cagcttgagggtctgaatc-3’	160
GAPDH	F: 5’-ctctgctcctcctgttcgac-3’<br>R: 5’-gcccaatacgaccaaatcc-3’	119

Supplemental Table 2. Western Blot antibodies and dilutions

Antibody	Catalog Number	Producer	Dilution
MEOX2	GTX55157	GeneTex, USA	1:3000
GLI-1	ab134906	Abcam, UK	1:1000
SMARCB1	GTX33509	GeneTex, USA	1:3000
EGFR	4267S	Cell Signaling Technology, USA	1:1000
CBP	GTX56255	GeneTex, USA	1:500
EZH2	GTX82503	GeneTex, USA	1:2000
BRD9	ab259839	Abcam, UK	1:1000
TWIST1	sc-81417	Santa Cruz, USA	1:500
MTA2	ab8106	Abcam, UK	1:1000
GAPDH	sc-47724	Santa Cruz, USA	1:3000
Rabbit IgG, HRP-conjugated	GTX213110-01	GeneTex, USA	1:10000
Mouse IgG, HRP-conjugated	GTX213111-01	GeneTex, USA	1:10000

Supplemental Table 3. *EGFR* gene regions specific primers.		
**EGFR region**	**Primer (F=forward; R=reverse)**	**Product length (bp)**
-51,774 to -51,648	F: 5’-gccaagtttcagacaagtgcc-3’<br>R: 5’-gtgaaaccctcctcctccaa-3’	126
-33,573 to -33,441	F: 5’-ctgggtttgtatgtgggcaa-3’<br>R: 5’-cgaagatgagagtggctgca-3’	132
-23,315 to -23,193	F: 5’-tggtaagctgagctgaagcc-3’<br>R: 5’-attggttctggcccactgag-3’	120
-336 to -235	F: 5’-ctcgacctggacataggctg-3’<br>R: 5’-gggtgccctgaggagttaat-3’	101
36,426 to 36,618	F: 5’-aaccgaacatgtgcgcattc-3’<br>R: 5’-tgggcaatcagtgtgagtca-3’	192
46,635 to 46,751	F: 5’-gagttcaagcgtccccagaa-3’<br>R: 5’-tgccatgcatgtcaaaagca-3’	116
55,382 to 55,501	F: 5’-atgtgggtttacagctggcc-3’<br>R: 5’-tctggaaagacttcggtggc-3’	119

Supplementary Table 4. *GLI-1* gene regions specific primers.		
**GLI-1 region**	**Primer (F=forward; R=reverse)**	**Product length (bp)**
-2192 to -2009	F: 5’-aggccgtgtgacatgtgatt-3’<br>R: 5’-gacagagcgagactccgtct-3’	183
-1830 to -1673	F: 5’-tcggactcctgacttgaggt<br>R: 5’-tcttctccccacccagttct-3’	157
-1541 to -1375	F: 5’-ccagcctgggcaaatagtga-3’<br>R: 5’-tcagagacccagctcagtca-3’	166
-822 to -665	F: 5’-ccctccagaacttcgagacg-3’<br>R: 5’-ggctctggaagaaggtgagg-3’	157
-612 to -457	F: 5’-ttccatccaaagggtgaggc-3’<br>R: 5’-ccccgacaaccagattgagg-3’	155

Supplemental Table 5. *SMARCB1* gene regions specific primers.		
**SMARCB1 region**	**Primer (F=forward; R=reverse)**	**Product length (bp)**
-429 to -248	F: 5'-catccgctgttgtcattttg-3'<br>R: 5'-tatctggaagcctgggtggt-3'	181
-228 to -128	F: 5'-cctcacttcgttgcttttcc-3'<br>R: 5'-cggacaaaaatagccaatga-3'	100
-41 to 111	F: 5'-gcccggccttttgtttg-3'<br>R: 5'-gaaggcgaaatgcggacc-3'	152
221 to 300	F: 5'-gctgagcaagaccttcggg-3'<br>R: 5'-ctcggagccgatcatgtagaa-3'	79

**Supplemental Table 6.** *EZH2* gene regions specific primers.		
**EZH2 region**	**Primer (F=forward; R=reverse)**	**Product length (bp)**
-759 to -604	F: 5'-ctcagcctcccgaagtagc-3'<br>R: 5'-gcctgtaatcccagcacttt-3'	155
-321 to -125	F: 5'-aggataggtggcgggaac-3'<br>R: 5'-ggttcaaacttgcttccag-3'	125
-18 to 52	F: 5'-cccaatcgccatcgctttta-3'<br>R: 5'-ctcgcgtcccgcctaatc-3'	70
224 to 400	F: 5'-ctcgagctgccaccctg-3'<br>R: 5'-gaaggagctgtgagcttcgg-3'	176
